# Supplementary material for: Medication Non-Adherence in Inflammatory Bowel Disease: A Systematic Review Identifying Risk Factors and Opportunities for Intervention
Source: Pharmacy (Basel). 2025 Feb 7;13(1):21. doi: 10.3390/pharmacy13010021 (PMC11859822; doi:10.3390/pharmacy13010021)
Supplement: Supplementary file 1 [file pharmacy-13-00021-s001.zip › FINAL Supplementary Table S1a and Table S1b_Full list of Search Terms.pdf]

***Supplementary Table S1a:* Key search terms**

Inflammatory Bowel Disease	**AND**	Medication	**AND**	Adherence
Inflammatory bowel disease **OR** IBD **OR** Crohn’s **OR** Ulcerative colitis **OR** Crohn*		Medic* **OR** Medicine* **OR** Treat* **OR** Therap* **OR** Drug* **OR** biologic* **OR** steroid* **OR** anti-TNF **OR** immunosuppress* **OR** Anti-inflammator* **OR** Salicylate* **OR** infusion* **OR** Disease manage* **OR** regime*		Adheren* **OR** Non-adheren* **OR** Complian* **OR** Non-complian* **OR** Concordan* **OR** Non-concordan* **OR** Persisten* **OR** Non-persisten*

Supplementary Table S1b: **Search strategy for all Databases**

Search	Query	Results
#S3	Inflammatory Bowel Disease/ **OR** IBD/ **OR** Crohn* disease/ **OR** Ulcerative colitis/ **OR** colitis/	
#S4	TIABSU("Inflammatory Bowel Disease") [TIABSU=title, abstract, subject data]	
#S4	TIABSU("IBD")	
#S4	TIABSU("Crohn* disease")	
#S4	TIABSU("Ulcerative colitis")	
#S4	TIABSU("Colitis")	
S6 – S14	MESH + Subject headings	
#S15	3 OR 4 OR 6 OR 7 OR 8 OR 9 OR 10 OR 11 OR 12 OR 13 OR 14	
#8	Medicine/**OR** Medication*/ **OR** Treat*/ **OR** Therap*/ **OR** Drug*/ **OR** biologic*/ **OR** Steroid*/ **OR** Immunosuppress*/ **OR** Anti-inflammator*/ **OR** Antiinflammator*/ **OR** Anti inflammator*/ **OR** Salicylate*/ **OR** Infusion*/ **OR** Disease manage*/ **OR** regime*/	
#9	TIABSU("Medic")	
#10	TIABSU("Treat*")	
#11	TIABSU("Therap*")	
#12	TIABSU("Drug*")	
#13	TIABSU("Biologic*")	
#14	TIABSU("Steriod*")	
#15	TIABSU("Immunosuppress*")	
#16	TIABSU("Anti-Inflammator*")	
#17	TIABSU("Anti inflammatory*")	
#18	TIABSU("Antiinflammator*")	
#19	TIABSU("Salicylate*")	
#20	TIABSU("Infusion*")	
#21	TIABSU("Disease manage*")	
#22	TIABSU("Regime*")	
MESH	Exact("Colitis, Ulcerative" OR "Inflammatory Bowel Diseases" OR "Crohn Disease")	
Subject headings	Exact("crohn disease" OR "inflammatory bowel diseases" OR "crohn disease" OR "colitis, ulcerative" OR "ulcerative colitis" OR "inflammatory bowel disease" OR "crohn's disease")	
	5 OR 16 OR 17 OR 18 OR 20	
#23	#8 OR 9# OR #10 OR #11 OR #12 OR #13 OR #14 OR #15 OR #16 OR #17 OR #18 OR #19 OR #20 OR #21 OR #22	
#24	Adheren* **OR** Non-adheren* **OR** Complian* **OR** Non-complian* **OR** Concordan* **OR** Non-concordan* **OR** Persisten* **OR** Non-persisten* OR drug adheren* OR medicat* adheren* OR complian* drug OR complian* medicat* OR medicat* complian* OR medicat* non adheren* OR medicat* non complian* OR medicat* non-adheren* OR medicat* non-complian*	

	OR medicat* nonadheren* OR medicat* noncomplian* OR medication persisten* OR non-adheren* medicat* OR non-complian* medicat* OR nonadheren* medicat* OR noncomplian* medicat* OR persisten* medicat*	
#25	TIABSU(“Adheren*”)	
#26	TIABSU(“Non-adheren*”)	
#27	TIABSU(“Complian*”)	
#28	TIABSU(“Non-complian*”)	
#29	TIABSU(“Concordan*”)	
#30	TIABSU(“Non-concordan”)	
#31	TIABSU(“Persisten*”)	
#32	TIABSU(“Non-persisten*”)	
	TIABSU(“nonpersistent*”)	
	TIABSU(“non persisten*”)	
	TIABSU(“nonconcordan*”)	
	TIABSU(“non concoran*”)	
	TIABSU(“noncomplian**”)	
	TIABSU(“non concordan*”)	
#33	Adheren*, drug	
#34	Adheren*, medication.mp	
#35	Complian*, drug.mp	
#36	Complian*, medication.mp	
#37	drug adheren*	
#38	drug complian*	
#39	medication adheren*	
#40	medication complian*	
#41	medication non adheren*	
#42	medication non complian*.mp	
#43	medication non-adheren.mp	
#44	medication non-complian.mp	
#45	medication nonadheren*.mp	
#46	medication noncomplian*.mp	
#47	medication persisten*.mp	
	Medication nonpersisten*	
	Medication non-persisten*	
	Medication non persisten*	
#53	#24 **OR** #25 **OR** #26 **OR** #27 **OR** #28 **OR** #29 **OR** #30 **OR** #31 **OR** #32 **OR** #33 **OR** #34 OR #35 OR #36 OR #37 OR #38 OR #39 OR #40 OR #41 OR #42 OR #43 OR #44 OR #45 OR #46 OR #47 OR #48 OR #49 OR #50 OR #52	
#54	#8 **AND** #23 **AND** #53	
#55	Exp “Systematic Review”/ **OR** Exp “Review”/ **OR** Exp“Intervention”/ **OR** Exp “Protocol”/ **OR** “Conference Abstract”/	
#56	Exp Clinical Trial Protocol/ or Clinical Trial Protocol.mp.	
#57	Exp Internet-Based Intervention/ or Internet-Based Intervention.mp. or (exp Psychosocial Intervention/ or Psychosocial Intervention.mp.)	
#58	54 NOT 55 NOT 56 NOT 57	

Limits:
-English Language,
-Human Participants,
- 16 years and above
