# Supplementary material for: Medication Non-Adherence in Inflammatory Bowel Disease: A Systematic Review Identifying Risk Factors and Opportunities for Intervention
Source: Pharmacy (Basel). 2025 Feb 7;13(1):21. doi: 10.3390/pharmacy13010021 (PMC11859822; doi:10.3390/pharmacy13010021)
Supplement: Supplementary file 1 [file pharmacy-13-00021-s001.zip › FINAL Supplementary Table S2_Participant Demographics.pdf]

Supplementary Table S2 Participant Demographics

Author, (Year), Country/ ies	Participants: 1) Number 2) Sex (%)	Aim / Specific Sample	Age: mean/ median/ mode, (R= range; SD)	Crohn's Disease/ Ulcerative Colitis (%)	Diagnosis length: Mean / Median/ %	Medication: 1) Class 2) Route (%)	Medication Regime/s/ Dosage	1) Smokers % 2) Consume alcohol	1) Employment status (%) 2) Education level: P (1°); S (2°); T (3°) 3) Relationship Status/ Living Status
Amiesimaka et al (2023), New Zealand	1) 7<br>2) F (85.7%)<br>M (14.3%)	Patients experiences to determine experiences factors influencing medication adherence, for the first time	Min: NR; (R= 20 – 70 yrs)	CD (71.4%)<br>UC (28.6%)	NR	1) NR<br>2) NR	NR	1) NR<br>2) NR	1) NR<br>2) NR<br>3) NR
Andrade et al (2020), Brazil	1) 302<br>2) F (62.9%)<br>M (37.1%)	Patients at a referral centre, receiving follow-up.	Mean: 45.8 yrs	CD (38.4%),<br>UC (61.6%)	NR	1) NR<br>2) NR	NR	1) NR<br>2) NT	1) NR<br>2) Illiterate (1%)<br>Literate *(read+write without education)* (21.6%)<br>P (18.6%)<br>Higher (44.5%)<br>University (14%)<br>Not declared (0.3%)<br>3) Married (45%)<br>Single (44.3%)<br>Divorced (6.7%)<br>Widower (4%)
Bager et al (2016), Denmark	1) 300<br>2) F (61%)<br>M (39%)	Outpatients with IBD	Median: 35 yrs	CD (50.6%)<br>UC (46.3%),<br>IUC (3%)	(%):<br>< 1 yr (15.6%),	1) All<br>2) NR	NR	1) NR<br>2) NR	1) NR<br>2) NR<br>3) NR

Author, (Year), Country/ies	Participants: 1) Number 2) Sex (%)	Aim / Specific Sample	Age: mean/ median/ mode, (R= range; SD)	Crohn's Disease/ Ulcerative Colitis (%)	Diagnosis length: Mean / Median/ %	Medication: 1) Class 2) Route (%)	Medication Regime/s/ Dosage	1) Smokers % 2) Consume alcohol	1) Employment status (%) 2) Education level: P (1°); S (2°); T (3°) 3) Relationship Status/ Living Status
					1-2 yrs (14.0%), 3-5 yrs (17.7%), >5 yrs (52.7%)				
Balaii et al (2018), Iran	1) 137<br>2) F (51.8%)<br>M (29.2%)<br>Unreported (19%)	Identifying barriers in patients with IBD who are non-adherent	Min: 15 years; Mean: 33.3yrs	CD (25.5%), UC (74.5%)	(%):<br>< 1 yr (19%),<br>1-5 yrs (34.3%),<br>>5 yrs (51.1%)	1) All<br>2) NR	NR	1) NR<br>2) NR	1) NR<br>2) Illiterate (0.7%)<br>P (10.2%)<br>High school (40.9%)<br>BSc (28.7%)<br>MSc (8%)<br>MD., PhD. (2.9%)<br>Other (8.8%)<br>3) Married (56.9%)<br>Single (41.6%)<br>Divorced (1.5%)
Ballester et al (2019), Spain	1) 274<br>2) F (50.7%)<br>M (49.3%)	Non-adhering patients with UC prescribed 5-ASA	Median: 38 yrs	UC (100%)	Median duration: 10 yrs	1) 5-ASA<br>2) Oral (72%)<br>Topical (5%)<br>Combined (23%)	OD,<br>Median dose: 3g/day	1) Active (14%)<br>Ex (31%)<br>Never (55%)<br>2) NR	1) NR<br>2) NR<br>3) NR
Banerjee et al (2021), India	1) 467<br>2) F (38.3%)<br>M (61.7%)	Indian patients with IBD	Mean: 38.6 yrs	CD (40.3%)<br>UC (59.7%)	Median duration: 28 months	1) All<br>2) All	NR	1) Smoker: 10.7%<br>2) Drink Alcohol: 16.5%	1) Employed (58.7%)<br>Unemployed (41.3%)<br>2) No schooling (5.3%)

Author, (Year), Country/ ies	Participants: 1) Number 2) Sex (%)	Aim / Specific Sample	Age: mean/ median/ mode, (R= range; SD)	Crohn's Disease/ Ulcerative Colitis (%)	Diagnosis length: Mean / Median/ %	Medication: 1) Class 2) Route (%)	Medication Regime/s/ Dosage	1) Smokers % 2) Consume alcohol	1) Employment status (%) 2) Education level: P (1°); S (2°); T (3°) 3) Relationship Status/ Living Status
									Secondary -high school (28.5%)<br>Tertiary-graduate or above (66.2%)<br>3) Married (80.7%)<br>Unmarried (19.3%)
Barnes et al (2021), Australia	1) 262<br>2) F (58%)<br>M (42%)	Medication adherence + CAM use during COVID-19 pandemic in patients with IBD	Median age: 46 yrs (IQR: 35-57)	CD (69%)<br>UC (28%)<br>IBDU (2%)	NR	1) All<br>2) All	NR; only prednisolone dose reported: <20mg / day (80%)	1) NR<br>2) NR	1) NR<br>2) NR<br>3) NR
Bhasin et al (2016), Canada	1) 765<br>2) F (53.2%)<br>M (46.8%)	To determine rates+ reasons for nonuse of IBD-specific medication in a referral clinic	Mean: 43.4 yrs<br>(SD= ±16.1)	CD (55.3%)<br>UC (44.7%)	Mean: 14.15 yrs	1) All<br>2) All	NR	1) NR<br>2) NR	1) NR<br>2) NR<br>3) NR
Billioud et al (2011), France	1) 108<br>2) F (64.8%)<br>M (35.2%%)	Adherence to adalimumab in patients with CD, reasons for non-adherence+ identify predictors for non-adherence	Min: >18 yrs;<br>Median: 35 yrs	CD (100%)	Median: 93.5 months	1) Biologics<br>2) Subcut (100%)	Data for 107 patients:<br>Induction regime:<br>-80/40mg (25.2%)<br>-160/80mg (74.8%)<br>Maintenance regimen:	1) Current (47.6%)<br>2) NR	1) NR<br>2) NR<br>3) Married (51.4%)<br>Divorced (4.8%)<br>Single (43.8%)

Author, (Year), Country/ ies	Participants: 1) Number 2) Sex (%)	Aim / Specific Sample	Age: mean/ median/ mode, (R= range; SD)	Crohn's Disease/ Ulcerative Colitis (%)	Diagnosis length: Mean / Median/ %	Medication: 1) Class 2) Route (%)	Medication Regime/s/ Dosage	1) Smokers % 2) Consume alcohol	1) Employment status (%) 2) Education level: P (1°); S (2°); T (3°) 3) Relationship Status/ Living Status
							-40mg every other week (67.6%)<br>-40mg weekly (18.5%)<br>80mg every other week (13.9%)		
Boyle et al (2015), USA	1) 70<br>2) F (44%)<br>M (56%)	Patients with UC prescribed rectal mesalamine	Min: ≥18 yrs;<br>Mean: 44 yrs	UC (100%)	Mean: 8 yrs	1) 5-ASA (Mesalamine)<br>2) Rectal	OD only reported:<br>Enema (61%)<br>Suppository (73%)	1) NR<br>2) NR	1) NR<br>2) NR<br>3) NR
Bruna-Barranco et al (2019), Spain	1) 181<br>2) F (45.9%)<br>M (54.1%)	Spanish patients with IBD	Min: 18 yrs;<br>Mean age: 47 yrs	CD (45.3)<br>UC (54.7%)	Mean: 10.21 yrs	1) All<br>2) All	NR	1) Current (17.7%)<br>Previous (44.8%)<br>Non-smoker (37.6%)<br>2) NR	1) NR<br>2) NR<br>3) NR
Bucci et al (2017), Italy	1) 151<br>2) F (46.4%)<br>M (53.6%)	Adherence to treatment in Italian IBD patients	Min: >18 yrs;<br>(R= 18-75 yrs)	CD (42.4%)<br>UC (57.6%)	Median (range): 1-5yrs	1) 5-ASA<br>2) Oral, IV and subcut (% NR for all)	NR	1) NR<br>2) NR	1) NR<br>2) NR<br>3) NR
Calloway et al (2017), USA	1) 246<br>2) F (61.8%)<br>M (38.2%)	Noncompliance to Anti-TNF in patients with IBD with depressive symptoms	Min: ≥18 yrs;<br>Mean: 37 yrs;	CD (70%)<br>UC (30%)	NR	1) Biologics (Anti-TNF)<br>2) NR	NR	1) Tobacco users (20%)<br>Others (80%)<br>2) NR	1) NR<br>2) NR<br>3) NR

Author, (Year), Country/ ies	Participants: 1) Number 2) Sex (%)	Aim / Specific Sample	Age: mean/ median/ mode, (R= range; SD)	Crohn's Disease/ Ulcerative Colitis (%)	Diagnosis length: Mean / Median/ %	Medication: 1) Class 2) Route (%)	Medication Regime/s/ Dosage	1) Smokers % 2) Consume alcohol	1) Employment status (%) 2) Education level: P (1°); S (2°); T (3°) 3) Relationship Status/ Living Status
			(R= 19-76 yrs)						
Calvo-Arbeloa et al (2020), Spain	1) 178<br>2) F (39.9%)<br>M (60.1%)	Adherence to patients with IBD collected 1 of the 3 drugs studied from hospital pharmacy	Min: >18 yrs;<br>Mean: 46.08yrs (SD= ±14.86)	NR (for 100% of patients)	NR (for total cohort)	1) Biologics: Adalimumab (76.4%), Golimumab (8.4%), Ustekinumab (15.2%)<br>2) Subcut	NR	1) NR (for total cohort)<br>2) NR	1) NR (for total cohort)<br>2) NR (for total cohort)<br>3) NR
Campos et al (2016), Portugal	1) 112<br>2) F (58.3%)<br>M (41.7)	Assess adherence to immunomodulators+ determine therapeutic non-adherence predictors	Min: ≥18 yrs;<br>Mean: 37.2 yrs (SD= ±11)	CD (62.5%)<br>UC (37.5%)	Median: 10.3 yrs	1) Immunomodulators<br>2) NR	Number of current IBD therapeutics:<br>1 (30.4%)<br>2 (43.8%)<br>3 (18.8%)<br>4 (7%)<br><br>Other concomitant maintenance IBD therapeutics:<br>-5-ASA per os (40.2%)<br>-5-ASA topical (15.2%)	1) Smoker (23.2%)<br>Ex-smoker (19.6%)<br>Non-smoker (54.5%)<br>Non-specified (2.7%)<br>2) NR	1)Employee (65.2%)<br>Unemployed (17.9%)<br>Student (11.6%)<br>Retired (4.5%)<br>Non-specified (0.8%)<br>2) Primary (8.9%)<br>High-school (51.8%)<br>University (31.3%)<br>Non-specified (8%)<br>3) Married (52.7%)<br>Divorced (8.9%)<br>Widow (0.09%)<br>Single (36.6%)<br>Non-specified (0.09%)

**Author, (Year), Country/ ies**	**Participants: 1) Number 2) Sex (%)**	**Aim / Specific Sample**	**Age: mean/ median/ mode, (R= range; SD)**	**Crohn's Disease/ Ulcerative Colitis (%)**	**Diagnosis length: Mean / Median/ %**	**Medication: 1) Class 2) Route (%)**	**Medication Regime/s/ Dosage**	**1) Smokers % 2) Consume alcohol**	**1) Employment status (%) 2) Education level: P (1°); S (2°); T (3°) 3) Relationship Status/ Living Status**
							-Infliximab (49.1%)		
Can et al (2022); Turkey	1) 253<br>2) F (44.8%)<br>M (55.2%)	Evaluating beliefs about treatment+ its effect on adherence to treatment in the Turkish population with IBD	Min: <18 yrs;<br>Mean: 44.75 yrs	CD (34%)<br>UC (66%)	67.05 months	1) NR<br>2) NR	Number of IBD drugs:<br>1 (44.9%)<br>2 (40.6%)<br>≥3 (6.85%)<br><br>Total daily drugs: 6.75<br><br>Daily medication frequency (%):<br>-Once (15.55%)<br>-Twice (20.6%)<br>-Thrice (40.5%)<br>- 4 times (23.4%)	1) 18.2%<br>2) NR	1) NR<br>2) Primary (51.3%)<br>3) NR
Coenen et al (2016), Belgium	1) 466<br>2) F (50.2%)<br>M (49.8%)	Identifying predictors of low adherence in the Belgian IBD population	Median: 42.5 yrs; (IQR= 31-53.3 yrs)	CD (71%)<br>UC (29%)	NR	1) All<br>2) All	NR	1) Smoker (21%)<br>Non-smoker (79%)<br>2) NR	1) Employed (51%)<br>Self-employed (8%)<br>Unemployed (6%)<br>Student (4%)<br>Retired (14%)<br>Incapacity (17%)<br>2) No schooling (7%)<br>Craft (17%)

Author, (Year), Country/ ies	Participants: 1) Number 2) Sex (%)	Aim / Specific Sample	Age: mean/ median/ mode, (R= range; SD)	Crohn's Disease/ Ulcerative Colitis (%)	Diagnosis length: Mean / Median/ %	Medication: 1) Class 2) Route (%)	Medication Regime/s/ Dosage	1) Smokers % 2) Consume alcohol	1) Employment status (%) 2) Education level: P (1°); S (2°); T (3°) 3) Relationship Status/ Living Status
									Secondary (34%)<br>Bachelor (28%)<br>University (14%)<br>3) Married (51%)<br>Living together (18%)<br>Not married (10%)<br>Single (21%)
Dasarathy et al (2023), USA	1) 410<br>2) F (72%)<br>M (28%)	Examining association between adherence to oral medications in patients with UC+ psychological distress, relationship with healthcare providers, motivation+ competence	Min: 18 yrs<br>Median: 49.5 yrs	UC (100%)	Median: 17.5 yrs	1) NR<br>2) Oral (100%)	Number of median medications (1)	1) NR<br>2) NR	1) NR<br>2) NR<br>3) NR
de-Castro et al (2017), Spain	1) 203<br>2) F (49%)<br>M (51%)	Assess adherence in patients with non-active IBD	Min: 18 yrs;<br>Mean: 46.3 yrs (SD = ±13.7)	CD (40%)<br>UC (60%)	Mean: 10.3 yrs	1) All<br>2) Topical or oral	OD (50%)<br>More than OD (50%)	1) Smoker (18.4%)<br>Non-smoker (81.5%)<br>(Of the 201 patients completing MMAS)<br>2) Only alcohol abuse recorded:<br>Yes (50%)<br>No (50%)	1) Employed (69.4%)<br>Not employed (30.6%)<br>(Of the 201 patients completing MMAS)<br>2) None or Primary studies (44.8%)<br>High school or university (55.2%)<br>(Of the 201 patients completing MMAS)

Author, (Year), Country/ ies	Participants: 1) Number 2) Sex (%)	Aim / Specific Sample	Age: mean/ median/ mode, (R= range; SD)	Crohn's Disease/ Ulcerative Colitis (%)	Diagnosis length: Mean / Median/ %	Medication: 1) Class 2) Route (%)	Medication Regime/s/ Dosage	1) Smokers % 2) Consume alcohol	1) Employment status (%) 2) Education level: P (1°); S (2°); T (3°) 3) Relationship Status/ Living Status
									3) Living with another (95.5%)<br>Not living with another (4.5%)<br>(Of the 201 patients completing MMAS)
Denesh et al (2021), UK	1) 298<br>2) F (51.3%)<br>M (48.7%)	Explore patients' preferences for route, form + frequency of IBD medication	Min: ≥18 yrs;<br>Median: 46 yrs	CD (48.3%)<br>UC (45.6%)<br>IBD-U (6%)	Median: 7 yrs	1) All<br>2) NR	NR	1) NR<br>2) NR	1) NR<br>2) NR<br>3) NR
Devlen et al (2014), USA	1) 27<br>2) F (52%)<br>M (48%)	Understand attitudes to adherence patients with IBD	Min:20 yrs; Mean: 31.5 yrs (SD= ±9)	CD (22%)<br>UC (78%)	Mean: 6.5 yrs	1) 5-ASA (Mesalamine)<br>2) Oral	OD (55%)<br>BD (33%)<br>TDS (12%)	1) NR<br>2) NR	1) NR<br>2) NR; although article states: *"Since a proportion of this population is frequently at college."*<br>3) NR; although article states: "*Since a proportion of this population is frequently beginning new relationships."*
Eindor-Abarbanel et al (2018), Israel	1) 311<br>2) F (62.4%)<br>M (37.6%)	Identify risk factors for non-adherence in IBD patients	Min: ≥18 yrs;<br>Median: 34.78 yrs	CD (70.4%)<br>UC (26%)<br>IBDU (3.5%)	NR	1) All<br>2) NR	NR *(only steroids categorised as low*	1) Current (14.6%)<br>Ex (11.04%)<br>Never (74.35%)	1) Employed (74.9%)<br>Unemployed (25.1%)<br>*(only data for 307 participants)*

Author, (Year), Country/ ies	Participants: 1) Number 2) Sex (%)	Aim / Specific Sample	Age: mean/ median/ mode, (R= range; SD)	Crohn's Disease/ Ulcerative Colitis (%)	Diagnosis length: Mean / Median/ %	Medication: 1) Class 2) Route (%)	Medication Regime/s/ Dosage	1) Smokers % 2) Consume alcohol	1) Employment status (%) 2) Education level: P (1°); S (2°); T (3°) 3) Relationship Status/ Living Status
							(<10,bg/day) or regular. Raw data NR)	*(only data for 308 participants)*<br>2) NR	2) Not university graduate (43.6%)<br>University graduate (56.4%)<br>*(only data for 307 participants)*<br>3) Single (27%)<br>In a relationship (66.1%)<br>Past relationship (6.8%)<br>*(only data for 307 participants)*
Engel et al (2017), Israel	1) 165<br>2) F (49%)<br>M (51%)	Whether re-phrasing question by HCP can help reveal more about patients who are non-adherent	Min: NR;<br>Mean: 33.7 yrs;<br>(SD= ±12.7)	CD (62.4%)<br>UC (29.6%)<br>IBDU (7.8%)	Mean: 9.13 yrs	1) All *(data inconsistent in Table1 and text)*<br>2) NR	Single medication (51.5%)	1) NR<br>2) NR	1) NR<br>2) P; 12 yrs (42.4%)<br>S; BA (39.4%)<br>High; MSc degree, PhD, MD (18.2%)<br>3) Single (46%)<br>Married (54%)
Franco et al (2022), Brazil	1) 90<br>2) F (50%)<br>M (50%)	To verify prevalence + influence of socio-demographic, clinical + pharmacotherapeutic characteristics	Min: >18 years<br>Mean: 50.51yrs<br>(SD= ±12.94)	UC (100%)	Up to 10 years (54.4%)<br>>10 years (45.6%)	1) All<br>(5-ASA, 93%; Immunosuppressants, 37.8%; Steroids, 13.3%)<br>(Patients may take more than 1 type)<br>2) NR	Daily pills:<br>-Up to 5/day (51.1%)<br>- >5/day (48.9%)<br><br>Total medications:	1) Smoking:<br>Yes (9.9%)<br>No (90.1%)<br><br>Alcoholism:<br>Yes (3.3%)<br>No (96.7%)	1) In activity (formal/informal employment) (30%)<br>No activity (unemployed/retired) 70%)<br>2) Illiterate (3.3%)<br>Literate (96.7%)

Author, (Year), Country/ ies	Participants: 1) Number 2) Sex (%)	Aim / Specific Sample	Age: mean/ median/ mode, (R= range; SD)	Crohn's Disease/ Ulcerative Colitis (%)	Diagnosis length: Mean / Median/ %	Medication: 1) Class 2) Route (%)	Medication Regime/s/ Dosage	1) Smokers % 2) Consume alcohol	1) Employment status (%) 2) Education level: P (1°); S (2°); T (3°) 3) Relationship Status/ Living Status
		associated with non-adherence to Tx of UC in remission					-1 medication (51.1%)<br>- >1 medication (48.9%)		3) Married/ consensual marriage (54.4%)<br>Single (45.6%)
Freitas et al (2015), Brazil	1) 147<br>2) F (57.1%)<br>M (42.9%)	To test possible independent associations of religious coping with clinically significant levels of medication adherence (+anxiety, depression +QoL) in adults diagnosed with IBD	Min: NR<br>Mean:45.1 yrs<br>(SD= ±14.08)	CD (43.5%)<br>UC (56.5%)	Mean: 80.5 months	1) NR<br>2) NR	NR	1) NR<br>2) NR	1) NR<br>2) Mean years of education (8.37)<br>3) Marital status with a spouse/ partner (66.7%)

Author, (Year), Country/ ies	Participants: 1) Number 2) Sex (%)	Aim / Specific Sample	Age: mean/ median/ mode, (R= range; SD)	Crohn's Disease/ Ulcerative Colitis (%)	Diagnosis length: Mean / Median/ %	Medication: 1) Class 2) Route (%)	Medication Regime/s/ Dosage	1) Smokers % 2) Consume alcohol	1) Employment status (%) 2) Education level: P (1°); S (2°); T (3°) 3) Relationship Status/ Living Status
Gallinger et al (2016), Canada	1) 204<br>2) F (100%)<br>M (0%)	Assess medication adherence during pregnancy in women with IBD	Min: 16 yrs;<br>Mean: 32.8 yrs<br>(SD= ±8.0)	CD (51.5%)<br>UC (31.9%)<br>IBDU (16.7%)	Mean: 2.85 yrs	1) All<br>2) NR	NR	1) NR<br>2) NR	1) Employed (65.7%)<br>Self-employed (8.3%)<br>Unemployed (1.5%)<br>Home-maker (8.8%)<br>Student (11.8%)<br>Disabled (3.9%)<br>2) Elementary school (1.0%)<br>High school (13.2%)<br>College/University (58.8%)<br>Postgraduate (27%)<br>3) NR
Gatapoulou et al (2021), Greece	1) 81<br>F (45.7%)<br>M (54.3%)	Assess impact of golimumab on health-related QoL+ other patient reported outcomes in patients with UC in Greece	Min: 18 yrs;<br>Median: 44.6yrs	UC (100%)	Median: 4 yrs	1) Biologic (Golimumab)<br>2) Subcut (100%)	Initial dose: 200mg,<br>Week 2: 100mg/week<br>Week 6:<br>50 or 100mg (dependent on body weight), then every 4 weeks	1) Current (7.4%)<br>Past (27.2%)<br>Never (65.4%)<br>2) NR	1) Employed (29.7%)<br>2) NR<br>3) NR

Author, (Year), Country/ ies	Participants: 1) Number 2) Sex (%)	Aim / Specific Sample	Age: mean/ median/ mode, (R= range; SD)	Crohn's Disease/ Ulcerative Colitis (%)	Diagnosis length: Mean / Median/ %	Medication: 1) Class 2) Route (%)	Medication Regime/s/ Dosage	1) Smokers % 2) Consume alcohol	1) Employment status (%) 2) Education level: P (1°); S (2°); T (3°) 3) Relationship Status/ Living Status
Ghadir et al (2016), Iran	1) 500<br>2) F (55%)<br>M (45%)	Evaluating non-adherence rate + most common related factors in patients with IBD in Iran	Min: 18 yrs;<br>Mean: 36.2 yrs; (R: 18-78)	CD (30%),<br>UC (68.2%),<br>IBDU (1.8%)	Mean: 7.67 years	1) NR<br>2) NR	NR	1) Current (12.4%)<br>Ex (6.2%)<br>Non-smoker (81.4%)<br>2) NR	1) Employed/Student/Collegian (61.4%),<br>Unemployed (38.6%)<br>2) Illiterate (3.2%)<br><12 yrs (13.8%)<br>>12yrs (39.6%)<br>License (32.2%)<br>Upper (10.4%)<br>3) Single (27.2%)<br>Married (69.8%)<br>Divorced (1.2%)<br>Widow/widowed (1.8%)
Gillespie et al (2014), UK	1) 58<br>2) F (44.8%)<br>M (55.2%)	Adults in remission with UC	Min: >18 yrs; Mean: 49.4 yrs; (SD= ±15.72)	UC (100%)	Median: 6.0 yrs	1) 5-ASA (Mesalazine, 100%)<br>2) Oral (100%)	OD (48.3%)<br>TDS (51.7%)	1) Current (10.3%)<br>Ex (44.8%)<br>Non-smoker (44.8%)<br>2) NR	1) Full-time (55.2%),<br>Not in full-time employment (44.8%)<br>2) NR<br>3) NR
Gomez-Medina et al (2022), Spain	1) 41<br>2) F (29.3%)<br>M (70.7%)	To quantify+ evaluate thiopurine prescription rate trends +determine impact+ risk of	Min: ≥18 yrs;<br>Mean: 44 yrs	UC (100%)	Mean: 11 yrs	1) Thiopurine<br>2) Oral	Median regimen: 3 tablets /day<br>Mean Azathioprine daily dose /kg: 1.79;	1) 9.8%<br>2) NR	1) NR<br>2) NR<br>3) NR

**Author, (Year), Country/ ies**	**Participants: 1) Number 2) Sex (%)**	**Aim / Specific Sample**	**Age: mean/ median/ mode, (R= range; SD)**	**Crohn's Disease/ Ulcerative Colitis (%)**	**Diagnosis length: Mean / Median/ %**	**Medication: 1) Class 2) Route (%)**	**Medication Regime/s/ Dosage**	**1) Smokers % 2) Consume alcohol**	**1) Employment status (%) 2) Education level: P (1°); S (2°); T (3°) 3) Relationship Status/ Living Status**
		non-adherence in adults in remission with UC					Mean Mercaptopurine daily dose /kg: 1.17		
Goodhand et al (2013), UK	1) 144<br>2) F (43.1%)<br>M (56.9%)	Young Adults vs Adults	Min: ≥16 yrs<br>*Young Adults*<br>Mean: 20 yrs; (range: 16-24 yrs);<br><br>*Adults*<br><Min: 25 yrs;<br>Mean: 40 yrs (range: NR)	*Young Adults:*<br>CD (59%)<br>UC (37%)<br>IBDU (4%)<br><br>*Adults:*<br>CD (58%)<br>UC (38%)<br>IBDU (4%)	*Young Adults*<br>Mean: 6.4 yrs;<br><br>*Adults*<br>Mean: 13.9 yrs	1) Thiopurine<br>2) Oral	Regimen:<br>*Young Adults:*<br>OD (51%)<br>BD (36%)<br>TDS (7%)<br>QDS (6%);<br><br>*Adults:*<br>OD (54%)<br>BD (39%)<br>TDS (6%)<br>QDS (1%);<br><br>Dosage:<br>*Young Adults*:<br>Mean Azathioprine daily dose /kg: 1.9;<br><br>Mean Mercaptopurine daily dose /kg: 0.9;	1) *Young Adults:*<br>Current (9%)<br>Ex (11%)<br>Non-smoker (80%);<br>*Adults:* Current (27%)<br>Ex (20%)<br>Non-smoker (55%)<br>2) Mean weekly intake (units):<br>*Young Adults:* 2.6<br>*Adults:* 4.3	1) *Young Adults:*<br>Employed (21%)<br>Unemployed (9%)<br>Student (70%)<br>Retired (0%)<br>*Adults:*<br>Employed (69%)<br>Unemployed (19%)<br>Student (1%)<br>Retired (11%)<br>2) *Young Adults:*<br>None (3%)<br>Secondary (29%)<br>A-level (50%)<br>Degree (19%)<br>*Adults:*<br>None (24%)<br>Secondary (16%)<br>A-level (22%)<br>Degree (38%)<br>3) *Young Adults:*<br>Single (93%)<br>Married/partner (7%)<br>Divorced/widowed (0%)

Author, (Year), Country/ ies	Participants: 1) Number 2) Sex (%)	Aim / Specific Sample	Age: mean/ median/ mode, (R= range; SD)	Crohn's Disease/ Ulcerative Colitis (%)	Diagnosis length: Mean / Median/ %	Medication: 1) Class 2) Route (%)	Medication Regime/s/ Dosage	1) Smokers % 2) Consume alcohol	1) Employment status (%) 2) Education level: P (1°); S (2°); T (3°) 3) Relationship Status/ Living Status
							Adults:<br>Mean Azathioprine daily dose /kg: 1.8;<br><br>Mean Mercaptopurine daily dose /kg: 0.9		*Adults:*<br>Single (35%)<br>Married/partner (57%)<br>Divorced/widowed (8%)
Govani et al (2018), USA	1) 6,048<br>2) F (54%)<br>M (46%)	Patients prescribed biologics in an insured cohort	Min: NR;<br>Mean: 41 yrs; (SD= ±15)	*ADA:*<br>CD (77.2%)<br>UC (22.8%)<br><br>*CZP:*<br>CD (99.4%)	NR	1)Biologics (Adalimumab, ADA, Certolizumab, CZP)<br>2) Subcut	*ADA Induction:*<br>Ratio of 2.33 days/injection;<br>*Maintenance:*<br>Ratio of 15 days/injection.<br><br>*CZP Induction:*<br>Ratio of 7 days/kit;<br>*Maintenance:*<br>Ratio of 30 days/kit.	1) NR<br>2) NR	1) NR<br>2) NR<br>3) NR
Hodgkins et al (2012),	1) 400	Patients	Min: ≥18 years,	UC (100%)	Mean range yrs:	1) 5-ASA<br>2) Oral	Raw data NR	1) NR<br>2) NR	1) Employed (52–67%)

Author, (Year), Country/ ies	Participants: 1) Number 2) Sex (%)	Aim / Specific Sample	Age: mean/ median/ mode, (R= range; SD)	Crohn's Disease/ Ulcerative Colitis (%)	Diagnosis length: Mean / Median/ %	Medication: 1) Class 2) Route (%)	Medication Regime/s/ Dosage	1) Smokers % 2) Consume alcohol	1) Employment status (%) 2) Education level: P (1°); S (2°); T (3°) 3) Relationship Status/ Living Status
Canada, Germany, USA, UK	2) F range (58-73%)<br>M range (27-42%)	with UC and their 5-ASA Tx preferences for mild-moderate long-term therapy, preventing relapse in 4 countries.	Mean: 32.1-47 yrs (across all countries)		(4.3%-14.6%)				2) Education after 18 yrs (range: 57-81%)<br>3) NR
Horvath et al (2012), Hungary	1) 592<br>2) F (53.8%)<br>M (46.2%)	Evaluate whether health-related QoL influences Tx adherence in IBD patients + influence of demographics, therapeutic modalities + non-adherence or QoL	Min 15 yrs; Mean: 38 yrs (R= 15-81 yrs)	CD (59.5%), UC (40.5%)	Mean: 9 yrs (0.1-51 yrs)	1) All (72.6% received 5-ASA; 24% corticosteroids; 52.9% immunomodulators; 10% received biologics regularly);<br>2) NR	NR	1) *CD:*<br>Active smokers (25.8%)<br>Ex (20.5%)<br>Non-smoker (53.8%)<br>*UC:*<br>Active smokers (12%)<br>Ex (27.4%)<br>Non-smoker (60.7%)<br>2) NR	1) *CD:*<br>"Intellectual workers" (57.4%)<br>Physical workers (42.6%);<br>*UC:*<br>"Intellectual workers" (61.3%)<br>Physical workers (38.7%)<br>2) None (3.4%)<br>P<br>(21.6%)<br>S (51.9%)<br>Higher (23.1%)<br>Degree (38%)<br>3) NR

Author, (Year), Country/ ies	Participants: 1) Number 2) Sex (%)	Aim / Specific Sample	Age: mean/ median/ mode, (R= range; SD)	Crohn's Disease/ Ulcerative Colitis (%)	Diagnosis length: Mean / Median/ %	Medication: 1) Class 2) Route (%)	Medication Regime/s/ Dosage	1) Smokers % 2) Consume alcohol	1) Employment status (%) 2) Education level: P (1°); S (2°); T (3°) 3) Relationship Status/ Living Status
Iborra et al (2021), Spain	1) 234<br>2) F (47%)<br>M (53%)	Patients treated with biologics	Min: NR; Mean/ Median: NR	CD (76%), UC (22%), IBDU (2%)	NR	1) Biologics<br>2) IV (50%), Subcut (50%)	Only stated patients on:<br>A dose-escalated schedule:<br>IV (16%)<br>Subcut (2%)<br>An optimized schedule:<br>IV (19%)<br>Subcut (8%)	1) 29%<br>2) NR	1) Employed (59%), Unemployed (12%), Student (3%), Retired /Disabled (26%)<br>2) NR<br>3) NR
Kamp et al (2019), USA	1) 61<br>2) F (90.2%)<br>M (9.8%)	Examining the relationship between social support+ self-management with emerging adults with IBD	Min: 18 yrs; Mean: 24.7 yrs	CD (63.9%), UC (36.1%)	Mean: 76.3 months	1)All<br>(36.1% 5-ASAs; 60.7% Biologics; 18% Corticosteroids; 26.2% Immunomodulators)<br>2) NR	NR	1) NR<br>2) NR	1) Full-time employed (49.2%)<br>Part-time employed (16.45)<br>Unemployed/ student (34.4%)<br>2) P (16.4%)<br>S (26.2%)<br>T (57.4%)<br>3) Single (77.1%), Married (22.9%)
Kamperidis et al (2012), UK	1) 189 *(Adult group data reported + separated from*	Adolescents+ adults prescribed thiopurines+ either:	Min:<br>>22 yrs (adults);<br>>13-21 yrs (adolescents)	CD (53%), UC (43%),	Mean: 11.5 yrs	1) Immunomodulaters (Thiopurines)	OD (80%)<br>BD (15%)<br>TDS (4%)<br>QDS (1%)	1) NR<br>2) NR	1) NR<br>2) NR<br>3) NR

Author, (Year), Country/ ies	Participants: 1) Number 2) Sex (%)	Aim / Specific Sample	Age: mean/ median/ mode, (R= range; SD)	Crohn's Disease/ Ulcerative Colitis (%)	Diagnosis length: Mean / Median/ %	Medication: 1) Class 2) Route (%)	Medication Regime/s/ Dosage	1) Smokers % 2) Consume alcohol	1) Employment status (%) 2) Education level: P (1°); S (2°); T (3°) 3) Relationship Status/ Living Status
	adolescents where possible) 2) F (41%) M (59%)	i) suspected non-adherence OR ii) lack of response	Mean: 38 yrs (adults) Mean: 18.7 yrs (adolescents)			2) NR			
Kawakami et al (2012), Japan	1) 242 2) F (50.4%) M (49.6%)	Japanese UC patients prescribed aminosalicylates	Min: >20 yrs; Mean: 40.4 yrs	UC (100%)	<5 yrs (61%), >5 yrs (39%)	1) 5-ASA 2)Oral	<BD (29.3%), >TDS (70.7%) Mean dosage: 3g/day	1) NR 2) NR	1) Full-time employed /Family-operated business (60.6%) Other (39.4%) 2) Junior-High/High School (14.9%) Beyond High school (85.1%) 3) Marital partner (43.4%) No marital partner (56.6%)
Kawakami et al (2014), Japan	1) 429 2) F (43.4%) M (56.6%)	Patients with UC with a high possibility of current non-adherence to aminosalicylates	≥20 yrs; Mean: 39.9 yrs	UC (100%)	<5 yrs (33.4%), >5 yrs (66.6%)	1) 5-ASA (Mesalazine) 2)Oral	<BD (50.3%), >TDS (49.7%) Mean dosage: 3.5g/ day	1) NR 2) NR	1) Full-time employed/ Family-operated business (56.6%) Other (43.4%) 2) Junior-High/ High School

Author, (Year), Country/ ies	Participants: 1) Number 2) Sex (%)	Aim / Specific Sample	Age: mean/ median/ mode, (R= range; SD)	Crohn's Disease/ Ulcerative Colitis (%)	Diagnosis length: Mean / Median/ %	Medication: 1) Class 2) Route (%)	Medication Regime/s/ Dosage	1) Smokers % 2) Consume alcohol	1) Employment status (%) 2) Education level: P (1°); S (2°); T (3°) 3) Relationship Status/ Living Status
									(48.2%)<br>Beyond High school (51.8%)<br>3) Married (41.5%), No marital partner (58.5%)
Kawakami et al (2017), Japan	1) 671<br>2) F (45.7%)<br>M (54.3%)	Patients with UC prescribed aminosalicylates	>20 yrs;<br>Mean: 40.2 yrs	UC (100%)	Mean: 8.9 yrs	1) 5-ASA<br>2) Oral	<BD (33.4%)<br>>TDS (66.6%)<br>Mean number of tablets/ day: 7.8	1) NR<br>2) NR	1) Full-time employed/ Family-operated business (58.1%)<br>Other (41.9%)<br>2) Junior-High/ High School (47.6%)<br>College or higher (52.4%)<br>3) Married (58%%)<br>No marital partner (42%)
Keil et al (2018), Czech Republic	1) 198<br>2) F (42.9%)<br>M (57.1%)	Rates of compliance with mesalazine in patients with UC	Min: >18 yrs;<br>(R= 18 - ≥70 yrs)	UC (100%)	NR	1) Used a medication containing Mesalazine<br>2) Oral (tablets or sachets)	Number doses /day:<br>1 (35.4%)<br>2 (44.4%)<br>3 (18.2%)<br>Other (2%)	1) NR<br>2) NR	1) Mostly manual (38.9%)<br>Mostly nonmanual (58.6%)<br>2) P (12.1%)<br>S (63.6%)<br>T (23.7%)<br>3) Married (57.1%)

Author, (Year), Country/ ies	Participants: 1) Number 2) Sex (%)	Aim / Specific Sample	Age: mean/ median/ mode, (R= range; SD)	Crohn's Disease/ Ulcerative Colitis (%)	Diagnosis length: Mean / Median/ %	Medication: 1) Class 2) Route (%)	Medication Regime/s/ Dosage	1) Smokers % 2) Consume alcohol	1) Employment status (%) 2) Education level: P (1°); S (2°); T (3°) 3) Relationship Status/ Living Status
									Unmarried (40.9%)<br>Others (2.0%)
Keller et al (2018), USA	1) NR<br>2) NR	Individuals with IBD during key reproductive periods	NR	IBD only (100% of patients)	NR	1) NR<br>2) NR	NR	1) NR<br>2) NR	1) NR<br>2) NR<br>3) NR
Kim et al (2016), Korea	1) 287<br>2) F (32.4%)<br>M (67.6%)	Assess adherence rates + IBD patients' beliefs + attitudes re: medication	Min NR; Mean: 38.3 yrs (SD= ±14.5)	CD (49.8%)<br>UC (50.2%)	Mean: 5.3yrs	1)All<br>(5-ASA, 88.7%; Azathioprine/6 MP, 46.1%; Biologics, 14.4%; Topical agent, 13.6%)<br>2) NR	"Mean number of prescribed medication: 3.5"	1) NR<br>2) NR	1) Working (61.2%)<br>Not working (38.8%)<br>-----<br>Working time amongst working patients:<br>Regular work (54.3%)<br>Part-time work (9.1%)<br>Other (36.6%)<br>2) Married (54.9%)
Lachaine et al (2013), Canada	1) 1681<br>2) F (56.6%)<br>M (43.4%)	Assess adherence to mesalamine Tx and potential determinants in mild-moderate UC	Min: NR; Mean: 55.3 yrs (SD= ±17.8)	UC (100%)	NR	1) 5-ASA (Mesalamine)<br>2) Oral (100%)	NR	1) NR<br>2) NR	1) NR<br>2) NR<br>3) NR
Lasa et al (2020), Argentina	1) 582<br>2) F (60.1%)<br>M (39.9%)	Determine inadequate adherence to oral and	Min: >18 yrs; Median: 37 yrs	CD (23.2%)<br>UC (73.7%)<br>IBDU (3%)	Median: 6 yrs (0.5-35)	1) 5 ASA / Thiopurines / Biologics<br>2) Oral	OD+	1) 12.71%<br>2) NR	1) NR<br>2) P (7.56%)<br>S (33.5%)<br>T (58.9%)

Author, (Year), Country/ ies	Participants: 1) Number 2) Sex (%)	Aim / Specific Sample	Age: mean/ median/ mode, (R= range; SD)	Crohn's Disease/ Ulcerative Colitis (%)	Diagnosis length: Mean / Median/ %	Medication: 1) Class 2) Route (%)	Medication Regime/s/ Dosage	1) Smokers % 2) Consume alcohol	1) Employment status (%) 2) Education level: P (1°); S (2°); T (3°) 3) Relationship Status/ Living Status
		parenteral therapies in patients with IBD from Argentina+ identify associated factors.	(R= 21-72);						3) Lives alone: 17.5%
Lee et al (2019), Korea	1) 259<br>2) F (43.6%)<br>M (56.4%)	Identify predictors of low adherence to oral 5-ASA in Koreans with UC	Min: >15 yrs;<br>Mean: 44±14 yrs	UC (100%)	Mean: 5.89 yrs	1) 5-ASA<br>2) Oral	OD (47.9%)<br>BD/TDS (52.1%)	1) Current smokers: 5.4%<br>2) Consumed alcohol: 45.2%	1) NR<br>2) P (5.4%)<br>S (42.1%)<br>T (52.5%)<br>3) Single/ never married: 27%<br>Married: 73%
Lee et al (2020), Canada	1) 230<br>2) F (100%)	Adherent women 1 year prior pregnancy, who delivered infant, ≥20 weeks gestation	NR	IBD only (100% of patients)	NR	1) All<br>2) All	On Monotherapy (27.5%)<br>On Polytherapy (72.5%)	1) NR<br>2) NR	1) NR<br>2) NR<br>3) NR
Lim et al (2020), Korea	1) 93<br>2) F (36.6%)<br>M (63.4%)	Investigate relationship between Tx adherence + disease-related knowledge in adolescents	Min: NR;<br>Mean: 18.6 (SD= ±3.7)	CD (83.9%)<br>UC (16.1%)	NR	1)All (However excluded from adherence analysis)<br>2) Oral	NR	1) NR<br>2) NR	1) NR<br>2) NR<br>3) NR

Author, (Year), Country/ ies	Participants: 1) Number 2) Sex (%)	Aim / Specific Sample	Age: mean/ median/ mode, (R= range; SD)	Crohn's Disease/ Ulcerative Colitis (%)	Diagnosis length: Mean / Median/ %	Medication: 1) Class 2) Route (%)	Medication Regime/s/ Dosage	1) Smokers % 2) Consume alcohol	1) Employment status (%) 2) Education level: P (1°); S (2°); T (3°) 3) Relationship Status/ Living Status
		(+paediatrics) with IBD							
Linn et al (2013), Netherlands	1) 68<br>2) F (62%)<br>M (38%)	Measure information recall by UBD patients+ investigate relationship between recall+ medication intake behaviour.	Min: NR:<br>Mean: 40.5 yrs; (SD= ±14.9)	CD (79.4%);<br>UC (19.1%);<br>Unknown: (1.5%)	Mean: 9.6 yrs	1) NR<br>2) All (Infusion, 26.6%;<br>Pills, 42.6%;<br>Injection, 36.8%)	NR	1) NR<br>2) NR	1) Employed (75%)<br>Not employed (25%)<br>2) Low (26.5%)<br>Moderate (35.3%)<br>High (38.2%)<br>3) Living: Alone (23.5%)<br>With partner (23.5%)<br>With partner+ child(ren) (23.5%)<br>With child(ren) (23.5%)<br>Other (17.6%)
Linn et al (2016), Netherlands	1) 99<br>2) F (62.6%)<br>M (37.4%)	Exploring whether patient satisfaction is related to adherence+ relation to beliefs	Min: NR<br>Mean: 41.66 yrs (SD= ±14.87)	Unclear/ not reported	Mean: 10.98 yrs	1) Immunosuppressives or biologics<br>2) NR	NR	1) NR<br>2) NR	1) NR<br>2) Low (24.24%)<br>Moderate (38.39%)<br>Higher (37.37%)<br>3) NR
Linn et al (2019), Netherlands	1) 107<br>2) F (58.8%)<br>M (40.2%)	Impact of online information-seeking on medication beliefs +adherence	Min: NR<br>Mean: 42.3 yrs (SD= ±15.3)	CD (69.2%);<br>UC (30.8%)	NR	1) Immunosuppressives or biologics<br>2) NR	NR	1) NR<br>2) NR	1) NR<br>2) Highly or moderately educated (79.3%)<br>Unreported (20.3%)<br>3) NR

Author, (Year), Country/ ies	Participants: 1) Number 2) Sex (%)	Aim / Specific Sample	Age: mean/ median/ mode, (R= range; SD)	Crohn's Disease/ Ulcerative Colitis (%)	Diagnosis length: Mean / Median/ %	Medication: 1) Class 2) Route (%)	Medication Regime/s/ Dosage	1) Smokers % 2) Consume alcohol	1) Employment status (%) 2) Education level: P (1°); S (2°); T (3°) 3) Relationship Status/ Living Status
Magalhaes et al (2014), Portugal	1) 138<br>2) F (58.7%)<br>M (41.3%)	Assess frequency of non-adherence to treatment in IBD	Min: 16 yrs;<br>Mean: 34.9 yrs	CD (55.8%)<br>UC (44.2%)	Mean: 4.65 yrs	1) All<br>2) Oral	NR	1) 21.2%<br>2) NR	1) Employed (80%)<br>Unemployed (20%)<br>2) Low/ Medium (36.2%)<br>High (63.8%)<br>3) NR
Martelli et al (2017), France	1) 162<br>2) F (46.3%)<br>M (53.7%)	Assess adherence to infliximab therapy in IBD patients + investigate reasons + predictors for non-adherence	Min: >18 yrs;<br>Mean: 35.4 yrs	CD (74.7%%)<br>UC (25.3%)	Mean: 9.6 yrs	1) Biologics (Infliximab)<br>2) NR	5mg/kg (80.3%)<br>10mg/kg (19.7%)	1) Active smoker: Yes (32.1%)<br>No (67.9%)<br>2) NR	1) NR<br>2) NR<br>3) Married (63.6%)<br>Single (36.4%)
Mitra et al (2012), USA	1) 1693<br>2) F (49.6%)<br>M (50.4%)	Assess association between adherence to oral 5-ASA + all-cause costs and healthcare utilisation among patients with UC	Min: ≥18 yrs;<br>Mean: 42.3 yrs (SD= ±12.8)	UC (100%)	NR	1) 5-ASA<br>2) Oral	NR	1) NR<br>2) NR	1) NR<br>2) NR<br>3) NR
Moradkhani et al (2011), USA	111<br>F (77%)<br>M (23%)	Investigating IBD-related knowledge with coping	Min: >18 yrs;	CD (65%)<br>UC (35%)	Mean: 9 yrs	1) NR<br>2) NR	NR	1) NR<br>2) NR	1) Employed (69%)<br>Unemployed (31%)<br>2) Not a student (63%)<br>Student (37%)

Author, (Year), Country/ ies	Participants: 1) Number 2) Sex (%)	Aim / Specific Sample	Age: mean/ median/ mode, (R= range; SD)	Crohn's Disease/ Ulcerative Colitis (%)	Diagnosis length: Mean / Median/ %	Medication: 1) Class 2) Route (%)	Medication Regime/s/ Dosage	1) Smokers % 2) Consume alcohol	1) Employment status (%) 2) Education level: P (1°); S (2°); T (3°) 3) Relationship Status/ Living Status
		strategies+ medication adherence	Mean: 31 yrs (SD= ±8.5)						3) Married (53%) Divorced (7%) Single (40%)
Moss et al (2014), USA	1) 106 2) F (56%) M (44%)	Developing a valid tool identifying adherence barriers + predicting those at risk of non-adherence to mesalamine	Min: NR; Mean: 42 yrs (SD= ±19)	UC (100%)	NR	1) 5-ASA (Mesalamine: Pentasa/ Asacol/ Asacol HD/ Lialda/ Apriso) 2) All	OD (38%) BD (38%) TDS (24%) Mean: 3.8g (SD = ±1.4)	1) NR 2) NR	1) NR 2) Graduate degree (66.6%) (no additional data reported) 3) NR
Mountifield et al (2014), Australia	1) 463 2) F (60%) M (40%)	Determining frequency + attitudinal predictors of overall medication non-adherence	Min: NR; Mean: 50.3 yrs	UC (100%)	NR	1) All 2) All	NR	1) Current smokers (13.93%) Previous smokers (31.23%) 2) NR	1) Currently employed (59.4%) 2) NR 3) Currently partnered (93.6%)
Nahon et al (2011), France	1) 1069 2) F (64.2%) M (35.8%)	Studying adherence to treatment+ socioeconomic+ psychological factors in a large cohort of patients	Min: NR; Mean: 43.6 yrs (SD= ±15.4)	CD (62.8%) UC (2.16%) IBDU (35.1%)	Mean: 13.35 yrs	1) All 2) NR	Complicated dosing regimen (3.9%) Large number of tablets (20.8%)	1) Present (14.4%) 2) NR	1) Working (59.2%) Unemployed (4.7%) Disabled (7.3%) Homemaker (3.4%) Student (6.5%) Retired (18.9%) 2) High school graduation or higher (69%) 3) Married (66%)

**Author, (Year), Country/ ies**	**Participants: 1) Number 2) Sex (%)**	**Aim / Specific Sample**	**Age: mean/ median/ mode, (R= range; SD)**	**Crohn's Disease/ Ulcerative Colitis (%)**	**Diagnosis length: Mean / Median/ %**	**Medication: 1) Class 2) Route (%)**	**Medication Regime/s/ Dosage**	**1) Smokers % 2) Consume alcohol**	**1) Employment status (%) 2) Education level: P (1°); S (2°); T (3°) 3) Relationship Status/ Living Status**
									Separated/Divorced (6.4%)<br>Single (24.8%)<br>Widowed (2.9%)
Nguyen et al (2016), Canada	1) 392<br>2) F (51%)<br>M (49%)	Determining CAM users vs non-users and the influence of this on adherence to medical therapy	Min: ≥18 yrs;<br>Mean: 36.3 yrs	CD (60%)<br>UC (40%)	Mean: 12.6yrs	1) All<br>2) All	NR	1) 38%<br>2) NR	1) Employed (69.3%)<br>2) NR<br>3) Married (50.7%)<br>Single/divorced/widowed (49.3%)
Ozturk et al (2023), Turkey	1) 251<br>F (52.6%)<br>M (47.4%)	Explore any relationship between drug compliance+ QoL among IBD patients	Min: >18 yrs;<br>Mean: 44.60 yrs (SD= ±1.39)	CD (59%)<br>UC (41%)	Mean: 6.81 yrs	1) All<br>2) Oral (64.9%)<br>Rectal (8%)<br>Oral+ rectal (23.5%)<br>Intravenous (5.6%)<br>Subcut (7.6%)	NR	1) Yes (23.5%)<br>No (73.3%)<br>Former smoker (3.2%)<br>2) Yes (6.4%)<br>No (92%)<br>Former drinker (1.6%)	1) Civil servant (19.1%)<br>Worker (7.6%)<br>Retired (15.1%)<br>Self-employed (35.9%)<br>Housewife (22.3%)<br>2) Literate (2.8%)<br>Primary+ secondary school (39.4%)<br>High school (18.8%)<br>University+ higher education (39%)<br>3) Married (77.3%)<br>Single (22.7%)
Pittet et al (2014), Switzerland	1) 512<br>2) F (51.6%)<br>M (48.4%)	To examine association between information-seeking activity	Min: NR (However Adult patients only);	CD (54.9%)<br>UC (45.1%)	Mean: 7 yrs	1) All<br>2) Oral, topical or intravenous (% NR)	1x drug (58.4%)<br>2x drugs (27.1%)	1) NR<br>2) NR	1) Full-time employed (38.5%)<br>Part-time employed (23.6%)<br>Unemployed (2.3%)

Author, (Year), Country/ies	Participants: 1) Number 2) Sex (%)	Aim / Specific Sample	Age: mean/ median/ mode, (R= range; SD)	Crohn's Disease/ Ulcerative Colitis (%)	Diagnosis length: Mean / Median/ %	Medication: 1) Class 2) Route (%)	Medication Regime/s/ Dosage	1) Smokers % 2) Consume alcohol	1) Employment status (%) 2) Education level: P (1°); S (2°); T (3°) 3) Relationship Status/ Living Status
		+ treatment compliance in IBD patients	Mean: 41 yrs (SD= ±14)				3x drugs (10.4%) >3x drugs (4.1%)		In training (11.7%) At home (7.2%) Retired (15.2% Missing value (1.4%) 2) Primary (11.1%) Secondary (66.2%) University (21.9%) Missing value (0.8%) 3) Married (50.8%) Not married (49.2%)
Ramos et al (2021), Spain	1) 154 2) F (45.5%) M (54.5%)	Assess adherence of self-administered subcut biologic medications, prescribed for IBD +identify risk factors for non-adherence	Min: NR; Mean: 36.7 yrs (SD= ±34-39)	CD (80%) UC (20%)	NR	1) Biologics 2) Intravenous + subcut (% NR)	To include induction or maintenance Tx regime with anti-TNF agent or anti-IL12/23 agent Indication for dose: based upon clinical judgement of the responsible physician	1) Current smoker (63.6%) 2) NR	1) NR 2) NR 3) NR
Ribaldone et al (2017), Italy	1) 376 2) F (43%) M (57%)	Studying adherence to therapy in a single centre, eliminating bias of a different	Only ranges reported. Median range: 31-50 yrs	CD (58.8%) UC (39.1%) IBDU (2.1%)	Only ranges reported. Median diagnosis length	1) 5-ASA (Mesalazine) 2) Oral (100%)	NR	1) NR 2) NR	1) NR 2) NR 3) NR

Author, (Year), Country/ ies	Participants: 1) Number 2) Sex (%)	Aim / Specific Sample	Age: mean/ median/ mode, (R= range; SD)	Crohn's Disease/ Ulcerative Colitis (%)	Diagnosis length: Mean / Median/ %	Medication: 1) Class 2) Route (%)	Medication Regime/s/ Dosage	1) Smokers % 2) Consume alcohol	1) Employment status (%) 2) Education level: P (1°); S (2°); T (3°) 3) Relationship Status/ Living Status
		relationship of trust with various doctors			range: 11-15 yrs				
Selinger et al (2013), Australia and UK	1) 356<br>2) F (58.45%)<br>M (41.55%)	To identify modifiable risk factors potentially for new adherence interventions	Min: ≥18 yrs;<br>Mean: 46.95 yrs	CD (43%)<br>UC (49.7%)<br>IBDU (3.7%)<br>Unknown (3.6%)	Only ranges reported. Median diagnosis length range: 1-5 yrs	1) All<br>2) NR	NR	1) NR<br>2) NR	1) Yes (59.95%)<br>No (40.05%)<br>2) Primary (2.4%)<br>Secondary (25.15%)<br>College (32.4%)<br>Bachelor (24.55%)<br>Master (16.6%)<br>PhD (2.3%)<br>Other (4.9%)
Severs et al (2017), Netherlands	1) 2612<br>2) F (56.6%)<br>M (43.4%)	To identify predictors for future (non)adherence in IBD	Min: ≥18 yrs;<br>Mean: 48.05 yrs	CD (59.6%)<br>UC (40.4%)	Median: 14.05 yrs	1) All<br>2) Rectal, 1.36%;<br>Oral, 77.03%;<br>Intramuscular, 11.9%;<br>Parenteral, 9.7%	Number of different types of medication/ person years only reported:<br>1 (67.8%)<br>2 (28.2%)<br>3 (4.00%)<br><br>Pills/ day for oral medication/ person years only reported:<br>1 (42.2%)<br>2 (25.6%)<br>3 (20.4%)<br>4 (7.22%)<br>5+ (4.58%)	1) Current smoker (15.05%)<br>Never (53.6%)<br>Ex (31.35%)<br><br>2) NR	1) NR<br>2) Low education (61.5%)<br>3) NR

Author, (Year), Country/ ies	Participants: 1) Number 2) Sex (%)	Aim / Specific Sample	Age: mean/ median/ mode, (R= range; SD)	Crohn's Disease/ Ulcerative Colitis (%)	Diagnosis length: Mean / Median/ %	Medication: 1) Class 2) Route (%)	Medication Regime/s/ Dosage	1) Smokers % 2) Consume alcohol	1) Employment status (%) 2) Education level: P (1°); S (2°); T (3°) 3) Relationship Status/ Living Status
Shah et al (2020), USA	1) 460<br>2) F (63.6%)<br>M (36.4%)	Identifying risk factors for non-adherence	Min: ≥18 years; Median: 38.5 yrs (IQR: 29-47)	CD (85.4%)<br>UC (14.6%)	Median: 7.5 yrs	1) Biologics<br>2) Subcut	NR	1) Current smoker (23%)<br>2) NR	1) NR<br>2) NR<br>3) NR
Stone et al (2021), USA	1) 112<br>2) F (71.4%)<br>M (28.6%)	Validating the MARS-5 and determining predictors of medication adherence	Mean: 42.9 yrs (SD= ±12.8)	CD (67.9%)<br>UC (28.6%)<br>IBDU (3.6%)	NR	1) All<br>2) All (Only some reported: Oral 5-ASA, 27.6%; Rectal 5-ASA, 8.9%; Infusion biologic, 36.6%; Oral steroid, 10.7%)	Number of OBD medications:<br>1 (29.4%)<br>2 (46.4%)<br>3 (≥24.1%)	1) Daily (11.7%)<br>Occasional (3.6%)<br>None (84.7%)<br>2) NR	1) NR<br>2) NR<br>3) NR
Suzuki et al, (2021), Japan	1) 101<br>2) F (40.6%)<br>M (53.5%)<br>No response (5.9%)	To determine which meal habits amongst UC patients influence adherence to oral 5-ASA regimens	Mode: Age group of 40s (24.8%)	UC (100%)	>10 yr History (47%)	1) 5-ASA<br>2) Oral (100%)	Dosing frequency:<br>OD (20.8%)<br>BD (20.8%)<br>TDS (40.6%)<br>≥QDS (1.0%)<br>No response (16.8%)	1) Yes (77.2%)<br>No (16.8%)<br>No response (5.9%)<br>2) NR	1) Full-time (44.6%)<br>Part-time (13.9%)<br>Student / unemployed (27.8%)<br>No response (13.9%)<br>2) NR<br>3) NR
Tae et al (2016), South Korea	1) 138<br>2) F (44.2%)<br>M (55.8%)	To investigate association between treatment non-	Mean: 39.7 yrs; (SD= ±14.4)	CD (43.5%)<br>UC (56.5%)	< 1 yr (17.4%)<br>1 – 5 yrs (42%)	1) All<br>2) NR	No of medication (median): 2.0	1) Smokers (15.25%)<br>2) Drinking alcohol (58.25%)	1) Full-time employment (70.5%)<br>2) Middle school (10.25%)

Author, (Year), Country/ ies	Participants: 1) Number 2) Sex (%)	Aim / Specific Sample	Age: mean/ median/ mode, (R= range; SD)	Crohn's Disease/ Ulcerative Colitis (%)	Diagnosis length: Mean / Median/ %	Medication: 1) Class 2) Route (%)	Medication Regime/s/ Dosage	1) Smokers % 2) Consume alcohol	1) Employment status (%) 2) Education level: P (1°); S (2°); T (3°) 3) Relationship Status/ Living Status
		adherence and patients' knowledge of the prescribed medication among individuals with IBD			5 yrs+ (40.6%)				High school (38.05%)<br>College graduates (51.7%)<br>3) Married (54.7%)<br>Bereaved (1.15%)<br>Single (44.2%)
Tomar et al (2019), India	1) 266<br>2) F (46.6%)<br>M (%) (53.4%)	To identify rates +predictors of non-adherence to medications in IBD	Mean: 38.5yrs; (SD= ±12.7)	CD (23.3%)<br>UC (76.7%)	Mean: 6.4 yrs	1) All<br>2) NR	Total number of drugs taking:<br>1 (40.6%)<br>2 (46.6%)<br>3 (10.5%)<br>4 (2.3%)	1)Current smoker (1.1%)<br>Ex-smoker (9.4%)<br>Never smoked (89.5%)<br>2)Current drinker (0.7%)<br>Ex-drinker (5.3%)<br>Never /occasional drinker (93.9%)	1) Profession/ semi-profession (14.3%)<br>Clerical/ shop-owner/farmer (20.7%)<br>Skilled worker/ semi-skilled worker (18.4%)<br>Unemployed (46.6%)<br>2) Illiterate (9.8%)<br>Middle school/ primary school certificate (17.7%)<br>Intermediate or post-high school diploma/ certificate (32.3%)<br>Graduate/ Postgraduate (28.9%)<br>Professional/ honours (11.3%)<br>3) NR
van der Have (2016), Netherlands	1) 128<br>F (56%)<br>M (44%)	Assessing risk factors for non-adherence +	Mean: 37 yrs; (SD= ±14)	CD (81%)<br>UC or IBDU (19%)	Median: 8 yrs;	1) Biologics<br>2) NR	NR	1) Smoker (20%)<br>2) NR	1) NR<br>2) Low education (63%)<br>3) NR

Author, (Year), Country/ ies	Participants: 1) Number 2) Sex (%)	Aim / Specific Sample	Age: mean/ median/ mode, (R= range; SD)	Crohn's Disease/ Ulcerative Colitis (%)	Diagnosis length: Mean / Median/ %	Medication: 1) Class 2) Route (%)	Medication Regime/s/ Dosage	1) Smokers % 2) Consume alcohol	1) Employment status (%) 2) Education level: P (1°); S (2°); T (3°) 3) Relationship Status/ Living Status
		examined association between adherence to anti-TNF agents (biologics) and loss of response							
Wang et al (2020), China	1) 446<br>2) F (41.7%)<br>M (58.3%%)	To develop machine learning models to help predict CD patients of non-adherence to azathioprine, assisting caregivers to streamlining intervention processes	Min: NR;<br>Mean: 31.5 yrs	CD (100%)	Mean: 4.8 yrs	1) 5-ASA<br>2) NR	Mean duration on maintenance Tx: 34.15 months;<br>Mean dosage (mg/d): 66.9	1) Smokers (mean 4.7%)<br>2) Alcoholism (mean 3.8%)	1) NR<br>2) P (3.7%)<br>S (10.5%)<br>High-school (29.3%)<br>College (45.25%)<br>Postgraduate (11.3%)<br>3) Married (48.1%)
Watanabe et al (2021), Japan	1) 68<br>2) F (100%)	Effect of medication non-adherence on clinical activity + pregnancy outcomes	(Age at conception)<br>Median: 32.7 yrs;<br>(R=24-42 yrs)	UC (100%)	Median: 9.60 yrs	1) All<br>2) NR	Higher dose group: Received ≥3.6g daily of Asacol+ ≥4.0g daily Pentasa;<br>Lower dose group: other Tx (nothing further reported)	1) Smoking 3 months prior pregnancy (11.76%)<br>2) NR	1) NR<br>2) NR<br>3) NR

Author, (Year), Country/ies	Participants: 1) Number 2) Sex (%)	Aim / Specific Sample	Age: mean/ median/ mode, (R= range; SD)	Crohn's Disease/ Ulcerative Colitis (%)	Diagnosis length: Mean / Median/ %	Medication: 1) Class 2) Route (%)	Medication Regime/s/ Dosage	1) Smokers % 2) Consume alcohol	1) Employment status (%) 2) Education level: P (1°); S (2°); T (3°) 3) Relationship Status/ Living Status
Wentworth et al (2018), USA	1) 365<br>2) F (60%)<br>M (40%)	Assess adherence to biologics + identify risks for non-adherence	Mean: 40.9 yrs;<br>(SD= ±14.1)	CD (82%)<br>UC or IBDU (18%)	<10 yrs (50%)<br>≥10 yrs (50%)	1)Biologics (Infliximab, Adalimumab, Certolizumab pegol, Vedolizumab)<br>2) Subcut, 43.6%; Intravenous, 56.4%	NR	1) Tobacco use (19%)<br>2) NR	1) NR<br>2) NR<br>3) NR
Yen et al (2012), USA	1) 5664<br>2) F (52.8%)<br>M (47.2%)	Evaluate use + risk of 5-ASA associated with non-persistence +non-adherence	Min: ≥18 yrs;<br>Mean: 48.3 yrs; (SD= ±15.4)	UC (100%)	NR	1) 5-ASA (all types)<br>2) Oral	NR	1) NR<br>2) NR	1) NR<br>2) NR<br>3) NR
Yoon et al (2017), South Korea	1) 322<br>2) F (32.3%)<br>M (67.7%)	Applied the IBD-Disability Index to a Korean population, to identify predictive factors influencing IBD-related disability	Mean: 39.7 yrs; (SD= ±14.2yrs)	CD (46%)<br>UC (54%)	Mean: 5.9 yrs	1) All<br>2) NR	NR	1) NR<br>2) NR	1) NR<br>2) NR<br>3) NR
Yu et al (2019), China	1) 342<br>2) F (40%)<br>M (60%)	Studying use of internet/social media in CD+	Mean: 41 yrs; (R= 37-45 yrs)	CD (100%)	NR	1) NR<br>2) NR	NR	1) NR<br>2) NR	1) NR<br>2) Illiterate (0.3%)<br>Primary (3%)

Author, (Year), Country/ ies	Participants: 1) Number 2) Sex (%)	Aim / Specific Sample	Age: mean/ median/ mode, (R= range; SD)	Crohn's Disease/ Ulcerative Colitis (%)	Diagnosis length: Mean / Median/ %	Medication: 1) Class 2) Route (%)	Medication Regime/s/ Dosage	1) Smokers % 2) Consume alcohol	1) Employment status (%) 2) Education level: P (1°); S (2°); T (3°) 3) Relationship Status/ Living Status
		impact on medication adherence							High school / Technical school (36%)<br>College (59%)<br>Postgraduate education (2%)<br>3) NR
Zand et al (2019), USA	1) 133<br>2) F (46.6%)<br>M (53.4%)	To develop a brief screening tool to identify non-adherence + reasons	Min: ≥18yrs;<br>Mean: 41.25 yrs	CD (50.4%)<br>UC (47.4%)<br>IBDU (2.3%)	NR	1) All<br>2) Subcut self-injection (20.3%)<br>Infusion (36.8%)<br>(Remaining NR)	NR	1) Current smoker (5.3%)<br>(Remaining NR)<br>2) NR	1) NR<br>2) Some high school (1.5%)<br>High school graduate (7.5%)<br>Some College (18.8%)<br>College Graduate (39.8%)<br>Post- College Degree (31.6%)<br>Other (0.75%)<br>3) Married (43.6%)<br>Remaining NR
Zelante et al (2014), Italy	1) 559<br>2) F (50.1%)<br>M (49.9%)	Analysing factors independently associated with adherence to medical therapy in patients with IBD	Min: ≥18 yrs;<br>Otherwise, only ranges reported:<br>Mean and Median range: 46-56 yrs;	CD (52.8%)<br>UC (47.2%)	Only ranges reported. Median diagnosis length range: ≥5 yrs	1) NR<br>2) Self-medication reported only: 69.9%	NR	1) NR<br>2) NR	1) NR<br>2) High school (76.25%)<br>University (23.75%)<br>3) NR

Abbreviations: ADA: Adalimumab; Anti-TNF: Anti-tumor Necrosis Factor; BD: Twice daily; CAM: Complementary and Alternative Medicine use; CD: Crohn's Disease; CZP: certolizumab; EEN: Exclusive enteral

nutrition; g: gram; IBDU: Indeterminate Colitis; IQR: Interquartile range; IV: intravenous; l-term: long-term; MARS-5: Medication Adherence Report Scale-5; Min: Minimum; N/A: Not applicable; NR: Not reported; OD: once daily; QoL: Quality of Life; SD: Standard deviation; Subcut: Subcutaneous; TDS: three times daily; TNF: tumor necrosis factor blockers; Tx: treatment; UK: United Kingdom; UC: Ulcerative Colitis; USA: United States of America; yrs: years; 5-ASA: Aminosalicylates;
