# Supplementary material for: Medication Non-Adherence in Inflammatory Bowel Disease: A Systematic Review Identifying Risk Factors and Opportunities for Intervention
Source: Pharmacy (Basel). 2025 Feb 7;13(1):21. doi: 10.3390/pharmacy13010021 (PMC11859822; doi:10.3390/pharmacy13010021)
Supplement: Supplementary file 1 [file pharmacy-13-00021-s001.zip › FINAL Supplementary Table S3_Study Design.pdf]

Supplementary Table S3 **Study Design**

Reference	Study Design	Data Type	Site Type	Single or Multi centre	Time period	Data collection method/s	P Value	Analysis	Adherence Theory used	Quality
Amiesimaka et al (2023), New Zealand	Focus group discussions	Qualitative	Voluntary, self-managed, peer support group for Crohn's+ Colitis via social media+ visiting gastroenterology outpatient clinics from local hospital (Dunedin)	NA	Within 1 month (Focus Groups lasting: 1 hour, 30minutes each)	Guided by a moderator	N/A	N/A	NR	High
Andrade et al (2020), Brazil	Cross-sectional	Quantitative	Referral centre	Single	14 months	Questionnaire + notes review	$P < 0.05$	UVA	NR	Medium
Bager et al (2016), Denmark	Cross-sectional	Quantitative	Tertiary hospital	Single	4 months	Electronic Questionnaire	$P < 0.05$	UVA	NR	Low
Balaii et al (2018), Iran	Cross-sectional	Quantitative	University hospital	Single	NR	Questionnaires (adherence, demographics/ clinical)	$P \leq 0.05$	UVA	NR	Low *(limited data reported)*
Ballester et al (2019), Spain	Longitudinal, observational cohort study	Quantitative	Tertiary centre, University hospital	Single	2 years	Clinical retrospective data collected from Spanish IBD registry; and prospective data from pharmaceutical electronic management programme	$P < 0.05$	UVA, MVA	NR	High

Reference	Study Design	Data Type	Site Type	Single or Multi centre	Time period	Data collection method/s	P Value	Analysis	Adherence Theory used	Quality
Banerjee et al (2021), India	Cross-sectional	Quantitative	Tertiary IBD clinic	Single	2 years	Questionnaires, interviews (to elicit clinical+ disease-related details), IBD registry, prospectively maintained database	P< 0.05	UVA, MVA	NR	High
Barnes et al (2021), Australia	Cross-sectional	Quantitative	2 x Tertiary IBD units	Multi	3 months	Online survey	P< 0.05	UVA	NR	Medium
Bhasin et al (2016), Canada	Cohort	Quantitative	IBD speciality clinic	Single	2 years	F:F clinic visit (demographic, disease, clinical and adherence data) via patient interview + health record review	P< 0.05	NR	NR	Low
Billioud et al (2011), France	Observational	Quantitative + Qualitative	4x University hospitals	Multi	21 months	At each clinic visit, patient was asked questionnaire, administered by treating Physician.	P< 0.05	UVA, MVA	NR	Medium
Boyle et al (2015), USA	Cohort -Focus groups	Quantitative + Qualitative	Specialist IBD clinic	Single	12 months	Focus group discussions	P< 0.05	UVA	Grounded Theory (qualitative data analysis)	Medium
Bruna-Barranco (2019), Spain	Cross-sectional	Quantitative	Adult tertiary clinic	Single	3 month	Questionnaire + Survey	P< 0.01 (UVA); P< 0.05 (MVA)	UVA, MVA	NR	High
Bucci et al (2017), Italy	Cross-sectional	Quantitative	IBD clinic, University hospital	Single	NR	F:F anonymous paper questionnaire in clinic	P< 0.05	UVA, MVA	NR	High

Reference	Study Design	Data Type	Site Type	Single or Multi centre	Time period	Data collection method/s	P Value	Analysis	Adherence Theory used	Quality
Calloway et al (2017), USA	Cross-sectional	Quantitative	Tertiary care IBD centre	Single	2 years	Retrospective chart review	P< 0.05	UVA, MVA (Cox Proportional Hazards regression)	NR	Medium
Calvo-Arbeloa et al (2020), Spain	Cross-sectional observational analysis study	Quantitative	Tertiary care hospital	Single	6 months	Questionnaire, retrospective pharmacy dispensing records data for prior 4 months +previous biological treatment history	P≤ 0.05	UVA, MVA	NR	High
Campos et al (2016), Portugal	Transversal prospective study	Quantitative	Tertiary centre, Gastroenterology Department, University Hospital	Single	6 months	Questionnaire collecting data on adherence, demographics, IBD, therapeutic complexity, belief in therapeutics and anxiety and depression	P< 0.05	UVA, MVA (logistic regression)	NR	High
Can et al (2022), Turkey	Cross-sectional	Quantitative	Gastroenterology outpatient clinics, University Faculty of Medicine	Single	3 months	Questionnaires + structured form administered by independent research nurse, prior to clinical examination	P≤ 0.05	UVA, MVA	NR	High
Coenen et al (2016), Belgium	Cross-sectional observational study	Quantitative	Tertiary IBD centre, University Hospital	Single	5 months	Electronic adherence +socio-economic questionnaires (given whilst in clinical waiting room)	P< 0.05	UVA, MVA	NR	High
Dasarathy et al (2023), USA	Cross-sectional	Quantitative	Existing members from a previous research cohort	NA	3 months	Existing members of the registry received an online follow-up survey including baseline	P< 0.1	UVA, MVA	Self-determination theory	High

Reference	Study Design	Data Type	Site Type	Single or Multi centre	Time period	Data collection method/s	P Value	Analysis	Adherence Theory used	Quality
			recruited from an online registry			questionnaires, adherence-associated factors+ additional psychosocial factors				
de-Castro et al (2017), Spain	Cross-sectional survey	Quantitative	IBD outpatient clinic, Department of Gastroenterol ogy	Single	6 months	F:F questionnaire completion in clinic+ demographic, social data collection, clinical activity + additional IBD-related data, 6months prior enrolment from electronic medical records + pharmacy databases	$P \leq 0.05$	UVA, MVA	NR	High
Denesh et al (2021), UK	Cross-sectional survey	Quantitative	Tertiary centre, Teaching hospital	Single	NR	F:F quantitative interview with a nurse + electronic medical records reviewed	$P < 0.05$	UVA	NR	Medium
Devlen et al (2014), USA	Interviews + Focus Groups	Qualitative	Tertiary IBD clinical site	Single	NR	1:1 interviews X 10 + F:F Focus groups X 4	NA	NA	NR	Medium
Eindor-Abaranel et al (2018), Israel	Cross-sectional survey	Quantitative	Outpatient clinics @ 3 University hospitals	Multi	19 months	Questionnaire completion (demographics, disease parameters, HCP relationship, psychological profile + adherence)	$P < 0.05$	UVA, MVA	Self-efficacy / salutogenesis theory *(Antonovsky (1987)*	Medium* *(Reporting unclear)*
Engel et al (2017), Israel	Cross-sectional survey	Quantitative	Outpatient clinic @ Tertiary centre	Single	NR	Questionnaire completion (demographics, prescribed Tx +adherence data,	$P < 0.05$	UVA, MVA	NR	Medium

Reference	Study Design	Data Type	Site Type	Single or Multi centre	Time period	Data collection method/s	P Value	Analysis	Adherence Theory used	Quality
						disease information, beliefs/perspective re: medication, disease + Tx)				
Franco et al (2022), Brazil	Cross-sectional, observational study	Quantitative	Medical Clinic of Gastroenterology of Center for IBD	Single	6 months	Outpatients attending routine appointment were invited to answer a semi-structured questionnaire+ variety of instruments, evaluating socioeconomic characteristics+ personal history, and medical records reviewed	P< 0.05	UVA, Pearson Chi-Squared test	NR	Medium
Freitas et al (2015), Brazil	Cross-sectional survey	Quantitative	Outpatient centre, Gastroenterology service of Hospital Universitario Walter Cantido (HUWC), Brazil	Single	NR	Participants completed a range of questionnaires. Clinical data collected on standardised form from review of medical records	P< 0.05	UVA, MVA (regression models)	NR	High
Gallinger et al (2016), Canada	Cross-sectional survey	Quantitative	Specialised Tertiary Care centre	Single	8 months	Self-administered paper-based, author-developed survey with closed ended questions	P<0.05	UVA, Chi-squared test, ANOVA	NR	Low* *(Reviewers' query)*

Reference	Study Design	Data Type	Site Type	Single or Multi centre	Time period	Data collection method/s	P Value	Analysis	Adherence Theory used	Quality
Gatapoulou et al (2021), Greece	Prospective Cohort	Quantitative	18 centres: 17 public/private hospitals+ 1 private practice)	Multi	12 months	Data collected at baseline, 6 and 12 months using a paper-case report form given to participants	P< 0.05	UVA, BVA, Wilcoxon Signed rank	NR	Medium
Ghadir et al (2016), Iran	Cross-sectional survey	Quantitative *(note: "interviews" were conducted but this was to complete questionnaire).*	Research Centres (GI clinics)	Multi	NR	Questionnaire + Interviews	P< 0.05	UVA, MVA (Factor Analysis)	NR	Medium
Gillespie et al (2014), UK	Prospective Cohort	Quantitative	Clinics, NHS	Multi	12 months	Pill count, self-report, MEMS, statistical methods	P < 0.05 "Statistically discernible" replaces "statistically significant"	MVA (2-level, generalised logistic mixed effects model)	NR	Medium
Gomez-Medina et al (2022), Spain	Observational, Retrospective cohort study	Quantitative + Qualitative (but qualitative data NR)	Tertiary-centre, University Hospital	Single	2 years	Electronic medical records reviewed	P< 0.05	UVA, MVA	NR	Low
Goodhand et al (2013), UK	Cross-sectional	Quantitative	Tertiary Adult and paediatric IBD centre	Single	7 months	Electronic patient records, "ACORN" database, blood tests, questionnaires, statistical methods	P< 0.05	UVA, MVA	NR	High
Govani et al (2018), USA	Retrospective Cohort	Quantitative	Health / Insurance Database	Multi	4 years	Electronic data extraction (including medical records, reported, side-	P< 0.05	UVA, MVA	NR	Medium

Reference	Study Design	Data Type	Site Type	Single or Multi centre	Time period	Data collection method/s	P Value	Analysis	Adherence Theory used	Quality
						effects, medication escalation + discontinuation records, pharmacy fills, claims + payments made)				
Hodgkins et al (2012), Canada, Germany, USA, UK	Cross-sectional, Discrete-Choice Experiment Survey (Conjoint Analysis)	Quantitative	Independent 3rd party patient recruitment service	Multi	NR (although symptom flare-ups reported over last 12months)	Web-based data collection (3 self-administered electronic surveys)	P< 0.05	Regression approach (Discrete Choice Experiment, Generalised Estimating Equations)	NR	Medium
Horvarth et al (2012), Hungary	Prospective study	Quantitative	6 X Tertiary centres	Multi	NR	In clinic (F:F)	P< 0.05	UVA, MVA (Factor Analysis)	NR	Medium
Iborra et al (2021), Spain	Cross-sectional, observational	Quantitative	Tertiary Outpatient centre, University Hospital	Single	2 months	Online + telephone	NR	NR	NR	Low
Kamp et al (2019), USA	Cross-sectional	Quantitative	NA (Social media used)	NA (Social media used)	2 months	Online	P< 0.05	UVA, MVA (logistic regression)	Individual and Family Self-management Theory	High
Kamperidis et al (2012), UK	Retrospective, observational study	Quantitative	Outpatient centre, NHS Trust	Single	30 months	Online database+ blood tests	P< 0.05	UVA, MVA	NR	High
Kawakami et al (2012), Japan	Cross-sectional survey	Quantitative	Outpatient clinic, University hospital	Single	5 months	F:F (in clinic) + medical records reviewed	P≤ 0.05	UVA, MVA (Explorat-ory Factor analysis)	NR	Medium

Reference	Study Design	Data Type	Site Type	Single or Multi centre	Time period	Data collection method/s	P Value	Analysis	Adherence Theory used	Quality
Kawakami et al (2014), Japan	Cross-sectional	Quantitative	Outpatient clinics, three urban hospitals, Japan	Multi	8 months	F:F (in clinic) + medical records reviewed	P< 0.05	UVA, Factor analysis	Health Belief model	Medium
Kawakami et al (2017), Japan	Cross-sectional survey	Quantitative	Outpatient clinics, four urban hospitals, Japan	Multi	3.5 yrs / 43 months	F:F (in clinic) + medical records reviewed	P< 0.20	UVA, MVA (Logistic regression+ Factor analysis)	NR	Medium
Keil et al (2018), Czech Repulic	Observational survey	Quantitative	Outpatients, three medical centres, Czech Republic	Multi	7 months	F:F (in clinic)	P< 0.05	UVA	NR	Low
Keller et al (2018), USA	Retrospective, observational	Qualitative	NA (Social media used)	NA (Social media used)	3 yrs	Online	NA	NA	NR	High
Kim et al (2016), Korea	Cross-sectional	Quantitative	Three tertiary hospitals in Korea	Multi	1 yr	Self-report questionnaires (in clinic), interviews + medical records reviewed	P < 0.05	MVA	Necessity-Concerns framework with 4 attitudes of accepting, ambivalent, indifferent or sceptical (Horne et al)	High
Lachaine et al (2013), Canada	Retrospective prescription + medical claims analysis	Quantitative	Pharmaceutica l services of the Quebec provincial health-plan database	NA (Pharmaceuti cal Services database)	6 yrs	Data on prescription claims obtained from database + evaluated	P< 0.05	UVA, MVA (Stepwise backward regression)	NR	High

Reference	Study Design	Data Type	Site Type	Single or Multi centre	Time period	Data collection method/s	P Value	Analysis	Adherence Theory used	Quality
Lasa et al (2020), Argentina	Cross-sectional	Quantitative + Qualitative	Referral centres	Multi	69 days	Online	P< 0.1	UVA, MVA (logistic regression)	NR	Medium
Lee et al (2019), Korea	Cross-sectional	Quantitative	University Hospitals	Multi	215 days / 7 months	F:F	P< 0.05	UVA, MVA	NR	High
Lee et al (2020), Canada	Retrospective, observational cohort	Quantitative	Health Databases	Multi	4 years	Online	P< 0.05	Chi-squared test, Bonferroni correction, E-value analysis	NR	Medium
Lim et al (2020), Korea	Cross-sectional	Quantitative	University Hospital	Single	"Summer" of 2018	F:F	P< 0.05	Independent t test, X^2, Fisher's Exact test	NR	Low
Linn et al (2013), Netherlands	Cross-sectional	Quantitative + Qualitative	6 x Hospitals	Multi	43 months	F:F + telephone	P< 0.15	UVA, MVA / Content analysis	Ley's Cognitive model	Medium
Linn et al (2016), Netherlands	Cross-sectional	Quantitative	6 x Hospitals	Multi	40 months	Telephone	NR	UVA, MVA	Necessity-Concerns Framework	Medium* *(Reporting unclear+ not all data reported)*
Linn et al (2019), Netherlands	Retrospective, longitudinal cohort study	Quantitative	6 x Hospitals	Multi	NR	F:F + telephone	NR	UVA, ANOVA, Bonferroni test	Necessity-Concerns Framework	Medium
Magalhaes et al (2014), Portugal	Prospective cross-sectional study	Quantitative	Gastroenterology Outpatient department, Hospital	Single	NR	F:F written interview (questionnaire completion)	P< 0.05	UVA (+binary logistic regression)	NR	Medium
Martelli et al (2017), France	Observational	Quantitative	2 x University Hospitals	Multi	2 months	F:F interviews + medical records reviewed	P< 0.05	UVA, MVA	NR	Medium

Reference	Study Design	Data Type	Site Type	Single or Multi centre	Time period	Data collection method/s	P Value	Analysis	Adherence Theory used	Quality
Mitra et al (2012), USA	Observational Cohort study	Quantitative	LifeLink database (patients listed within)	NA	99 months	Review of "LifeLink" database prescription claims by UC patients between June 1997-August 2005	NR	UVA, MVA	NR	High
Moradkhani et al (2011), USA	Exploratory cross-sectional study	Quantitative	Online survey	NA	3 months	Electronic messages sent via online support/advocacy groups, with study survey link to access+ complete	P< 0.05	UVA, *(Only MVA for clinic-demographics variables + not adherence)*	NR	Medium
Moss et al (2014), USA	Prospective Observational study	Quantitative	Tertiary referral centre	Single	18 months	F:F questionnaire completion, urine sample collection + medical records + patient pharmacy records reviewed	P< 0.05	UVA, MVA	NR	High
Mountifield et al (2014), Australia	Cross-sectional questionnaire	Quantitative + Qualitative	3 x care settings in 2 distinct locations (Teaching Hospital IBD service; Gastro Private practice; Public Hospital in remote location).	Multi	NR	Patients identified from IBD databases/hospital records + mailed a questionnaire, with reminders sent 1 + 3 months	P< 0.05	UVA, MVA	NR	High
Nahon et al (2011), France	Retrospective Cohort	Quantitative	Mailed to all members of Association Francois Aupetit (French IBD patients' association) + accessible on	NA	7 months	Demographics collected via self-report questionnaire including asking patients to assess adherence to Tx during previous 4 weeks by a Visual Analogue Scale.	P< 0.05	UVA, MVA (Stepwise logistic regression)	NR	Medium

Reference	Study Design	Data Type	Site Type	Single or Multi centre	Time period	Data collection method/s	P Value	Analysis	Adherence Theory used	Quality
			association's website							
Nguyen et al (2016), Canada	Prospective Cohort study	Quantitative	Outpatient clinics at the IBD centre	Single	19 months	F:F Questionnaire completion, followed by follow-up questionnaire completion @ either 6/12 months in clinic, by phone or by email	NR	UVA, MVA	NR	High
Ozturk et al (2023), Turkey	Descriptive, Cross-sectional study	Quantitative	Medical Faculty Hospital Gastroenterology Department Inflammatory Bowel Disease Polyclinic	Single	4 months	Self-report method	P< 0.05	Correlation analysis, Chi-squared test, Mann Whitney U test.	NR	Medium
Pittet et al (2014), Switzerland	Cross-sectional	Quantitative + Qualitative	Data from the Swiss IBD cohort study+ from a qualitative survey	Multi	34 months	Self-administered questionnaire sent to patients @baseline, with qualitative survey conducted in October 2009 with patients enrolled up to that time, investigating sources/themes of information	NR	UVA, MVA (logistic regression)	NR	Medium
Ramos et al (2021), Spain	Retrospective Cohort	Quantitative	Tertiary level hospital	Single	45 months	Clinical history review	P< 0.05	UVA, MVA (logistic regression, chi-squared analysis)	NR	High
Ribaldone et al (2018), Italy	Blind prospective	Quantitative	Outpatients tertiary clinic	Single	33 weeks	Self-administered, anonymous questionnaire,	P< 0.05	UVA, MVA	NR	Medium

Reference	Study Design	Data Type	Site Type	Single or Multi centre	Time period	Data collection method/s	P Value	Analysis	Adherence Theory used	Quality
						disease activity evaluated by Physician+ medical records reviewed				
Selinger et al (2013), Australia and UK	Prospective Cross-sectional	Quantitative	Outpatients from tertiary and secondary IBD clinics + outpatient offices of Gastroenterologists (Australia)	Multi	NR	Self-administered questionnaire	NR	UVA, MVA (Stepwise multiple linear regression)	NR	High
Severs et al (2017), Netherlands	Prospective cohort	Quantitative	University Medical Centers + General Hospitals	Multi	NR (but patients followed for 2.5 yrs)	Web-based baseline questionnaire followed by 3 monthly questionnaires	P= 0.157	UVA, MVA (logistic regression)	Common Sense Model	High
Shah et al (2020), USA	Retrospective Cohort analysis	Quantitative	Tertiary Outpatient clinic	Single	4 years	Electronic medical records + prescription claims data from speciality pharmacy database reviewed	P< 0.05	UVA, MVA	NR	High
Stone et al (2021), USA	Longitudinal	Quantitative	(No site, but participants recruited from previous longitudinal cohort study, Regional gastroenterology clinics, a provincial research registry, IBD Clinical and Research Centre	Multi	1 year	Online survey	Not reported	UVA, MVA (for missing data + logistic regression analysis)	NR	High

Reference	Study Design	Data Type	Site Type	Single or Multi centre	Time period	Data collection method/s	P Value	Analysis	Adherence Theory used	Quality
			website +study adverts in local hospitals +gastroenterologists' offices)							
Suzuki et al (2021), Japan	Cross-sectional	Quantitative	6 x Pharmacies (where patients received prescribed medications following outpatient appointments at University Hospital)	Multi	4 months	Self-reported, anonymous questionnaire completed in pharmacies	P<0.05	UVA, MVA	NR	Medium
Tae et al (2016), South Korea	Prospective Observational Cohort study	Quantitative	University Medical Centre Hospital	Single	7 months	Baseline completion of a self-administered questionnaire	P< 0.05	UVA, MVA	NR	High
Tomar et al (2019), India	Cross-sectional, Observational study	Quantitative + Qualitative	IBD Clinic of Department of Gastroenterology, All India Institute of Medical Sciences	Single	5 months	Interviews with researcher for questionnaire completion	P< 0.01	UVA, MVA (binary logistic regression)	NR	Medium
van der Have et al (2016), Netherlands	Observational	Quantitative	3x University Hospitals+ 3x general hospitals	Multi	12 months	Baseline questionnaire completion by patients, then prospectively followed up for 12 months+ local pharmacies contacted re: medication refills during follow-up+ electronic medical records reviewed	P< 0.10	UVA, MVA (logistic regression)	NR	High

Reference	Study Design	Data Type	Site Type	Single or Multi centre	Time period	Data collection method/s	P Value	Analysis	Adherence Theory used	Quality
Wang et al (2020), China	Cross-sectional	Quantitative	Patient of Gastro-Intestinal department of Shanghai hospital, affiliated to University School of Medicine, Shanghai	Single	13 years	Questionnaires of adherence, medication beliefs, medication knowledge+ anxiety+ depression provided to patients. Demographics, socioeconomics, clinical presentations, laboratory tests, therapeutic regimes+ follow-up records obtained from electronic medical records reviewed. Data added to a data for CD management	P< 0.05	UVA, MVA (multivariate logistic regression)	NR	High
Watanabe et al (2021), Japan	Prospective, Observational Cohort study	Quantitative	Specialised Gastroenterology hospitals	Multi	4 years	Access to registry of pregnant women to identify potential participants; clinical records reviewed + questionnaire completion 30 days preconception, each pregnancy trimester+30 days after delivery	P< 0.20	UVA, MVA (logistic regression analysis)	NR	High
Wentworth et al (2018), USA	Retrospective Cohort	Quantitative	University centre	Single	2 years	Pharmacy + infusion centre records reviewed	P< 0.05	UVA, MVA	NR	High
Yen et al (2012), USA	Retrospective Cohort	Quantitative	Insurer Database	Multi	4.5 years	Socio-demographics, health insurance information, existing comorbid conditions, clinical variables taken from health plan claims database	NR	UVA, MVA	NR	High

Reference	Study Design	Data Type	Site Type	Single or Multi centre	Time period	Data collection method/s	P Value	Analysis	Adherence Theory used	Quality
Yoon et al (2017), South Korea	Cross-sectional	Quantitative	4 IBD centres	Multi	6 months	Recruited at outpatient clinic visits + asked to complete 2 x questionnaires	P< 0.05	UVA	NR	Medium
Yu et al (2019), China	Cross-sectional	Quantitative	8 IBD centres in 6 large urban hospitals	Multi	137 days	Online questionnaires	P≤ 0.05	UVA, MVA	NR	Medium* *(Reporting unclear)*
Zand et al (2019), USA	Cross-sectional	Quantitative + Qualitative (but data NR)	Single University hospital	Single	6 months	Questionnaire completion via email or during clinic visits + medical records reviewed	P< 0.05	UVA, MVA	NR	High
Zelante et al (2014), Italy	Cross-sectional	Quantitative+ Qualitative (but data NR)	Patient society (Inflammatory Bowel Disease Association of Emilia-Romagna Region, Italy).	NA	NR	Anonymous questionnaire completion	P< 0.05	UVA, MVA (logistic regression)	NR	Medium* *(Reporting unclear)*

Abbreviations: ANOVA: Analysis of Variance; BVA: Bivariate analysis; CD: Crohn's Disease; F:F: face to face; GI: Gastrointestinal; IBD: Inflammatory Bowel Disease; IFSMT: Individual and Family Self-management Theory; NA: Not applicable; NR: Not reported; min: minute; MVA: Multivariate analysis; Tx: treatment; UC: Ulcerative Colitis; UK: United Kingdom; USA: United States of America; UVA: Univariate analysis; yr: year; yrs: years.

Key: *Quality of study re-reviewed, with reason stated.

Note: Terminology used is written as reported in each paper.
