# Supplementary material for: Medication Non-Adherence in Inflammatory Bowel Disease: A Systematic Review Identifying Risk Factors and Opportunities for Intervention
Source: Pharmacy (Basel). 2025 Feb 7;13(1):21. doi: 10.3390/pharmacy13010021 (PMC11859822; doi:10.3390/pharmacy13010021)
Supplement: Supplementary file 1 [file pharmacy-13-00021-s001.zip › FINAL Supplementary Table S4_Non-adherence results.pdf]

Supplementary Table S4 **Categorisation and Measuring Non-Adherence**

Reference	Terminology used	Measure of non-adherence	Cut off for non-adherence (Quantitative studies)	% of non-adherence/ adherence
Amiesimaka et al (2023)	**Adherence/ non-adherence, poor adherence**	**Self-report in Group Discussion**	***NA***	**NA**
Andrade et al (2020)	**Adherence/ nonadherence**	**Morisky Green Levine Scale – 4 item scale (MMAS-4)**	***Nonadherence:*** *≥1 positive response to 4 questions*	**72.7%** non-adherence
Bager et al (2016)	**Adherence/ non-adherence**	**Study Specific electronic Self-report questionnaire**	***Non-adherence: no cut off***<br>***Good adherence: reported consumption of 81 – 100% prescribed medication.***<br>***Other unnamed categories:***<br>***-50-80%***<br>***-0-49%***	**7%** considered not good adherence
Balaii et al (2018)	**Adherence/ non-adherence**	**Morisky Medication 8 item Adherence Scale (MMAS)**	*Scores ranged from: 0-8*<br>***Low adherence;*** *scores: <6;*<br>***Medium adherence;*** *scores: 6-7;*<br>***High adherence;*** *scores: 8*	**46%** low adherence
Ballester et al (2019)	**Adherence/ non-adherence**	**MPR** (% of prescribed doses of the 5-ASA that the patient was dispensed at the pharmacy, calculated by Pharmaceutical electronic management programme).	***Non-adherent:***<br>*-Patients who picked up <80% of prescribed medication from pharmacy*	**41%** non-adherent
Banerjee et al (2021)	**Adherence/ nonadherence**	**MMAS - 8 item**	*Scores ranged from: 0-8*<br>***Low adherence;*** *scores: <6;*<br>***Medium adherence;*** *scores: 6-7;*<br>***High adherence;*** *scores: 8*<br><br>***Nonadherence:*** *defined as MMAS score <6*	**51%** non-adherence
Barnes et al (2021)	**Adherence/ nonadherence**	**MARS – 10 item**	*Self-report scores are weighted and added to provide a score that allows classification into:*<br>***-non-adherent***<br>***-partially adherent***<br>***-adherent***<br>*(No cut-off stated).*	**3%** low adherence

Bhasin et al (2016)	**Use/ Non-use**	**NR**	*Clinician recorded judgement only (no questions specified).*	**18.8%** nonadherence
Billioud et al (2011)	**Adherence/ nonadherence**	**Direct questioning at clinic by Physician** (patients systematically asked:<br>- whether or not they delayed injecting / missed injection altogether over past 3 months?<br>If yes, they were asked:<br>-How many days this was delayed?<br>**-**Give reasons for their nonadherent behaviour?	***Nonadherent:*** *If an injection was missed delayed at least once, in 3 months prior. (An injection was considered "missed" if it was delayed by:*<br>*- 7+ days for patients receiving adalimumab every 1x week or*<br>*-14+ days for patients receiving adalimumab every 2 weeks*	**45.4%** patients reported missing or delaying at least one injection over the 3 months prior to the study;<br><br>**14.8%** patients reported missing at least one injection over the 3 months prior to the study
Boyle et al (2015)	**Adherence/ nonadherence**	**MPR**<br><br>Also refill calculations as 2° endpoints:<br>**-continuous single-interval medication availability**<br>**-continuous multiple-interval medication gap**	***Adherent:*** *>0.6 MPR*<br>***Non-adherent:*** *<0.6 MPR*	**71%** nonadherent
Bruna-Barranco et al (2019)	**Adherence/ nonadherence**	**MMAS - 8 item**	*Scores ranged from: 0-8*<br>***Low adherence;*** *scores: <6;*<br>***Medium adherence;*** *scores: 6-7;*<br>***High adherence;*** *scores: 8*	**22.7%** low adherence
Bucci et al (2017)	**Adherence/ nonadherence**	**MMAS - 8 item**<br><br>Also:<br>**- Study Specific Self-report 5-point Likert Scale** grading importance of taking medications,<br>**-Study Specific Self-report questionnaire:** frequent missing IBD drug	*Scores ranged from: 0-8*<br>***Low adherence;*** *scores: <6;*<br>***Medium adherence;*** *scores: 6-7;*<br>***High adherence;*** *scores: 8*	**23.24%** overall low adherence
Calloway et al (2017)	**Primarily Compliance/ noncompliance, but also adherence/ nonadherence for other research**	**Prescription completion/ receiving infusion.**	***Interruption of medication*** *(e.g. not completing an anti-TNF prescription (injectable) OR not getting an infliximab infusion for 30 days after due date).*	**13%** noncompliant

Calvo-Arbeloa et al (2020)	**Adherence/ non-adherence and Compliant/ noncompliant both used**	**MMAS - 4 item**<br><br>Also:<br>**- MPR**	***NR for MMAS-4 however typically:*** **Nonadherence:** ≥1 positive response to 4 questions.<br><br>***For MPR: Low adherence/ Scarcely compliant:*** ≤85% MPR.	**25.3%** low-adherence (whole cohort).<br>**46.9%** noncompliant (subgroup of low adherers).
Campos et al (2016)	**Adherence/ nonadherence**	**MMAS - 8 item**<br><br>Also:<br>**-Therapeutic Complexity**	*Questions relate to adherence to drugs in the previous 2 weeks. Scores ranged from: 0-8:*<br>***Low adherence;*** *scores: ≤5;*<br>***Medium adherence;*** *scores: 6-7;*<br>***High adherence;*** *scores: 8*<br>***Low adherence is split further into 2 categories:***<br>***-Intentional non-adherence:*** *majority of responses indicate behaviours not related to forgetfulness (e.g. stopped taking drugs without telling the doctor because they felt worse when they took it);*<br>***-Unintentional non-adherence:*** *majority of answers indicate behaviours related to forgetfulness.*<br><br>***Therapeutic complexity*** *defined by the challenges in adherence to drugs.*<br>*Higher scores indicate a greater level of treatment difficulty as perceived by the patient. A drug was complex /difficult to take, when result was greater than the scale mid-point.*	**29.5%** were non-adherent to immunosuppression.<br>**31.6%** were nonadherent to monotherapy;<br>**27.3%** nonadherent to combination therapy with thiopurines+ infliximab.<br>**57.6%** of patients were not intentionally nonadherent.<br><br>**42.4%** reported some degree of voluntary non-adherence;<br>**82.7%** sometimes forgot to take their IBD drugs.<br><br>*Therapeutic complexity (reported in qualitative table).*
Can et al (2022)	**Adherence/ non-adherence, low adherence**	**MARS-5**	***NR***	**36%** low /non-adherence overall; 41.9% in Ulcerative Colitis, 24.4% in Crohn's Disease.<br>**24.9%** Intentional non-adherence.<br>**24.1%** unintentional non-adherence
Coenen et al (2016)	**Adherence/ nonadherence + low adherence**	**MMAS - 8 item**	*Scores ranged from: 0-8. (Note: scores are opposite to typical scoring for MMAS-8):*<br>***Low adherence;*** *scores: >2;*	**36%** low adherers

			Medium adherence;** scores: 1-2;*<br>High adherence;** scores: 0*	
Dasarathy et al (2023)	**Adherence/ non-adherence**	**MMAS - 8 item**	*Scores ranged from: 0-8*<br>***Low adherence;** scores: ≤5;*<br>***Medium adherence;** scores: 6-7;*<br>***High adherence;** scores: 8*	**29%** participants had low adherence to oral UC medications (MMAS-8 score <6)
de-Castro et al (2017)	**Adherence/ non-adherence**	**MMAS - 8 item**<br><br>**MPR** (% of prescribed days drug supply obtained by patient during specific period/over period of refill intervals).	***<u>MMAS-8</u>***<br>*Scores ranged from: 0-8*<br>***Low adherence;** scores: ≤5;*<br>***Medium adherence;** scores: 6-7;*<br>***High adherence;** scores: 8*<br>***Medium + High adherers = grouped as good adherers***<br><br>***<u>MPR</u>***<br>***Non-adherent: <80%***<br>***Adherent: ≥80%%***	**22.4%** non-adherence (MMAS-8)<br><br>**37%** (MPR)
Denesh et al (2021)	**Adherence/ non-adherence**	**MARS-4**	***Non-adherence:** MARS score = ≤16*<br>***Adherence:** MARS score = >16*	**NR**
Devlen et al (2014)	**Adherence/ nonadherence**	**NA**	*NA*	**88.9%** reported intermittent non-adherence
Eindor-Abarbanel et al (2018)	**Adherence/ nonadherence**	**MMAS-8 item**	***<u>MMAS-8</u>***<br>*Scores ranged from: 0-8*<br>***Low adherence;** scores: <6;*<br>***Medium adherence;** scores: 6-7;*<br>***High adherence;** scores: 8*<br>***Medium + High adherers = grouped as good adherers***	**40.5%** low adherence
Engel et al (2017)	**Adherence/ non-adherence, compliance/ non-compliance also used interchangeably**	**Study Specific Self-report questionnaire**	***Non-adherence:*** taking <80% of prescribed medication doses.<br>*<u>Missed doses for:</u>*<br>*<u>-IV biologics:</u> a skipped infusion +/or delay in receiving IV infusion of above 7 days.*	**30.3%** not adherent

			-Subcut biologics: *a skipped injection +/or delay above 3 days in drug injecting.*	
Franco et al (2022)	**Adherence/ Non-adherence**	**MMAS-8**	***MMAS-8***<br>*Scores ranged from: 0-8*<br>***Low adherence;*** *scores: <6;*<br>***Medium adherence;*** *scores: 6-7;*<br>***High adherence;*** *scores: 8*<br>***Medium + High adherers = grouped as good adherers***	**77.8%** was the prevalence of non-adherence
Freitas et al (2015)	**Adherence/ nonadherence**	**MMAS-8**	***MMAS-8***<br>*Scores ranged from: 0-8*<br>***Low adherence;*** *scores: <6;*<br>***Medium adherence;*** *scores: 6-7;*<br>***High adherence;*** *scores: 8*<br>***Medium + High adherers = grouped as good adherers***	**NR**
Gatapoulou et al (2021)	**Adherence/ discontinuation**	**MPR**	***MPR***<br>***Discontinuation adherence rate: proportion of patients who prematurely discontinued golimumab treatment due to any reason.***<br><br>*(**Adherence rate:** calculated by dividing total number of golimumab injections dispensed with the scheduled number of golimumab injections over 12 months)*	**Non-adherence** not reported,<br>**Discontinuation:** 35.8% discontinued golimumab
Gallinger et al (2016)	**Adherence/ Non-adherence or stopped/ discontinued IBD medications**	**Study Specific Self-report questionnaire**	***Patients rated their adherence during current /previous pregnancies to IBD medications.***<br>***Any discontinued drug/s was asked whether this was based upon advice of clinician.***	**19.8%** non-adherence/ stopping
Ghadir et al (2016)	**Non-adherence:** "If patient stops or used their drugs less for any reason without physician's advice" OR "not using drugs according to physician's direction properly."	**Study Specific Self-report questionnaire**	***NR***	**33.3%** non-adherence

Gillespie et al (2014)	**Adherence: "High adherence," "low adherence/ not adherent"**	**Adherence:**<br>**- Study Specific Self report questionnaire**<br><br>**-Pill Count**<br><br>**-MEMs**	***Non-adherence:***<br>**-Self report:** *Taken study tablets as prescribed below 90% of the time.*<br>-**Pill Count:** *Number of tablets taken expressed as % of the correct number of tablets taken.*<br>***-MEMs:*** *% of days a participant was adherent (e.g. % of days a participant opened their bottle, the correct number of times)*	**10.7%** low / not-adherent (self-report);<br><br>(Only median % reported for:<br>-Pill Count: 3.3%<br>-MEMs: 10.8%)
Gomez-Medina et al (2022)	**Adherence/ non-adherence**	**MPR:** *calculated by percentage of prescribed doses of thiopurines the patient had delivered at the pharmacy/number of days of medication collected by patient.*	***Non-adherent:***<br>*-Patients who picked up <80% of prescribed medication from pharmacy.*	**22%** non-adherent.
Goodhand et al (2013)	**Non-adherence**	**-Thiopurine metabolite measurements**<br>**-MMAS-8**	***<u>Thiopurine metabolite measurements:</u>***<br>***Adherence:***<br>*6-TGN<100 pmol/RBC x10⁸, in the absence of a metabolite profile suggesting hypermethylation of thiopurines to MMP (MMP: 6-TGN >11) when patients are adequately dosed.*<br>***Partial non-adherence or under-dosing:***<br>*6-TGN levels of 100-199 pmol/RBC x 10⁸*<br><br>***<u>MMAS-8:</u>***<br>*Scores ranged from: 0-8*<br>***Low adherence;*** *scores: <6;*<br>***Medium adherence;*** *scores: 6-7;*<br>***High adherence;*** *scores: 8*<br>*(Statistical analysis was used to detect intentional non-adherence for scores of <6).*	**12%** non-adherent.
Govani et al (2018)	**Adherence/ non-adherence**	**MPR:** *calculated by summing days of medication supplied /sum of days in total refill intervals.*	***<u>ADA:</u>***<br>***Adherent:*** *>0.86 MPR*<br>***Non-adherent:*** *≤0.86 MPR*<br><br>***<u>CZP:</u>***<br>***Adherent:*** *>0.87 MPR*<br>***Non-adherent:*** *≤0.87 MPR*	**<u>Overall:</u>**<br>**24%** were below optimal level of adherence.<br><br>**<u>ADA:</u>**<br>**20.5%** non-adherent.<br>**<u>CZP:</u>**<br>**43.7%** non-adherent.

Hodgkins et al (2013)	**Adherence/ poor or non-adherence**	**MMAS 6-item** (non-specific)	***Good adherence:*** Total scores of 4-6 on the MMAS (non-IBD specific version)<br>***Poor adherence:*** Total scores of 0-3 on the MMAS (non-IBD specific version)	Average poor adherence rate: **39%;**<br>(Overall average adherence rate: **60%)**
Horvath et al (2012)	**Adherence/ non-adherence**	**Medication Adherence report (23 items)**	***Adherence:*** Taking more than or equal to 80% of prescribed medication<br>***Non-adherence:*** Taking less than 80% of prescribed medication	**13.4%** non-adherence overall
Iborra et al (2021)	**Adherence: "high adherence"**	**Review of treatment schedule, changes in IBD medication, dates of administration, cause of delay & telephone survey.**	***NR***	**7.15%** overall nonadherence.<br>**10%** *of infusion group non-adherent;*<br>**4.3%** *of subcut group non-adherent*
Kamp et al (2019)	**Adherent/ nonadherent**	**MARS-4**	***Scores ranged from: 4-20:***<br>***Low adherers* (=0);** *scores: 4-16;*<br>***High adherers* (=1);** *scores: 17-20*	**26.2% nonadherence**
Kamperidis et al (2012)	**Nonadherence or partial nonadherence**	**Thiopurine metabolite testing**	***Nonadherence:*** *Thiopurine levels <100pmol /red blood cell x 10^8 in absence of metabolite profile (suggesting shunting of thiopurines to MMP, rather than to 6-TGN if adequately dosed).*<br>*Partial nonadherence: Thiopurine levels 100-199pmol/red blood cell x 10^8*	**8%** *of adult group nonadherent.*<br>**29%** *adolescent group nonadherent.*<br>**12%** *overall nonadherent*
Kawakami et al (2012)	**Adherence/ nonadherence**	**Study Specific Self-administered 12 i**tem questionnaire with 5-point ordinal scale, specific focus on:<br>i) Difficulties in taking aminosalicylates;<br>ii) Disease state; iii) Psychosocial Factors.<br><br>(Plus additional 11 item scale **Trust in Physician Scale**).	***No cut-off for non-adherence***<br><br>(Trust in Physician Scale; higher score = greater trust).	**NR**
Kawakami et al (2014)	**Adherence/ non-adherence**	**Study Specific Self-administered 5-point Likert Scale questionnaire,** relating to 5	***Taking <80% of prescribed dose***	**29.6%** non-adherent

		domains of HBM. Higher score = lower beliefs.<br><br>**Question asked 7 days prior enrolment** re:<br>a) prescribed dose of ASA,<br>b) missed tablets.<br>a-b with the figure divided by prescribed dose (as per medical record) x 100, to create percentage.		
Kawakami et al (2017)	**Adherence/ non-adherence**	**Study Specific Self-administered 5-point Likert Scale**, with higher scores indicating more frequent use<br><br>**Reviewing 7 days prior enrolment** and the self-reported taken dose divided by the prescribed dose (as per medical record).<br>**-Pill count** over ~2 months.	Consumption of ≤80% of prescribed dose.	**27.7%** non-adherent
Keil et al (2018)	**Adherence/ non-adherence, compliance/ noncompliance**	**Study Specific Self-report questionnaire** covering:<br>-Medication Preference / Ideal dosage scheme,<br>- Forgetting,<br>-Quantity of Usage	Usage of <80% of medications	Noncompliance found in **21.2%** patients
Keller et al (2018)	**Qualitative study (NA)**	**NA**	***NA***	**NA**
Kim et al (2016)	**Adherence/ non-adherence**	**MARS**	***Scores ranged from: 4-20***<br>***Non-adherers****; scores: 4-16;*<br>***Adherers****; scores: 17-20*	**22.3%** did not adhere to medication
Lachaine et al (2013)	**Adherence/ non-adherence**	**MPR**	***Adherent* =** If patient had MPR ≥80% for a 5ASA Tx, estimated over 1 yr period.<br>Proportion of patients with a MPR of ≥50% was also calculated.	**72.3%** not adherent
Lasa et al (2020)	**Adherence: "Inadequate adherence"**	**Study Specific Self-report questionnaire**	***Answering * "Inadequate adherence":*** *indicates patients who answered "rarely," "sometimes," "often" or*	**50.3%** inadequate adherence

			"always" to the question "How often do you miss medication intake?"	
Lee et al (2019a)	**Adherence: low, medium, high.**	**MMAS-8**	***Low scores**: <6;*<br>***Medium scores**: 6-7;*<br>***High scores**: 8*	**49.8%** low adherence
Lee et al (2020)	**Adherence / non-adherence**	**MPR**	***Adherence:** MPR ≥80%*<br>***Non-adherence:** MPR <80%*	**12.6%** of women adherent 1 year prior pregnancy, were not adherent during pregnancy;<br>**40%** of women were either not adherent 1 year prior pregnancy or during pregnancy;<br>**13.2%** of women adherent 1 year prior pregnancy, discontinued medication during pregnancy.
Lim et al (2020)	**Adherence: "Good adherence"/ "Poor adherence."**	**Study Specific Self-report on amount of medications taken since previous clinic visit** *(validated by Medic, expressed in %)*.	***Good adherence:*** ≥80%<br>***Not good/poor adherence:*** <80%	**68.8%** "good adherence";<br>**31.2%** "poor adherence"*
Linn et al (2013)	**Adherence: "Very poor" - "Very good."**	**Study Specific Single, self-report item, Likert scale, 1 – 10.**	***Very poor:*** 1<br>***Very good*:**10	**9.1 / 91%** mean score (quite highly adherent), **54.4%** completely adherent.
Linn et al (2016)	**Adherence/ Non-adherence**	**MARS-5**	***A maximum cumulative score: 25***<br>***-Fully adherent***:25<br>***-Not fully adherent:*** <25	**30.30%** not fully adherent
Linn et al (2019)	**Adherence/ Non-adherence**	**MARS-5**	***Higher score indicates higher self-reported medication non-adherence***	NR
Magalhaes et al (2014)	**Adherence/ non-adherence, poor adherence**	**Study Specific Self-report questionnaire consisting of 23 questions regarding:** *Demographics, disease character, treatment data, adherence to treatment, frequency of forgetting medication, number*	***Non-adherent**:* if patient forgot at least a dose a week, often or very often, during last 12 months	**29.7%** reported non-adherence

		of doses usually forgotten/ week during preceding year and reason, smoking habits, and complementary and alternative medicine use.		
Martelli et al (2017)	**Adherence/ non-adherence, poor adherence**	**Systematically asked** whether they had delayed/ missed a perfusion of infliximab since beginning of Tx<br>(+ for delays, number of days between planned day of injection and real day, was recorded).	***Non-adherent:*** any delay/ missing at least one infliximab perfusion since beginning of treatment	**54.3%** overall non-adherence rate (including missing/delayed treatment)
Mitra et al (2012)	**Adherence/ nonadherence**	**MPR** (calculating a standard measure of medication refill pattern over time).	***Nonadherence MPR:*** Patients with a MPR of <0.8 (<80% adherence).<br>*Nonadherence was further sub-divided into groups of:*<br>*-60-79% nonadherence*<br>*-40-59% nonadherence*<br>*-20-39% nonadherence*<br>*-<19% nonadherence*	**72%** patients overall were nonadherent to 5-ASA treatment.<br><br>**18%** were nonadherent 60-79% of time;<br>**17%** were nonadherent 40-59% of time;<br>**17.2%** were nonadherent 20-59% of time;<br>**19.7%** were nonadherent 0-19% of time.
Moss et al (2014)	**Adherence/ nonadherence**	**MMAS-8**<br><br>**MPR** (calculating a standard measure of medication refill pattern over time).<br><br>**Study specific developed measure:**<br>10 item survey	***Low adherence;*** *scores: <6;*<br><br>***MPR: <0.6 / <60%***<br>*Described as "Patients with low MPR are typically taking fewer pulls per day over an extended period than prescribed.*<br>*(Other refill calculation measures were recorded as 2° endpoints: continuous single-interval medication availability, continuous multiple interval medication gap).*<br><br>***Study specific measure:*** *5-point Likert scale correlated with MPR and MMAS-8 results*	**32.5%** overall relative risk of non-adherence (in patients not strongly agreeing with adherence beliefs in questions 6 + 10, which were significantly associated with refill patterns)
Moradkhani et al (2011)	**Adherence/ non-adherence, low adherence, compliance,**	**MMAS-4**	***Low adherence:*** *scores 3-4;*	**NR**<br>(Mean Morisky score: 1.69, suggesting high adherence overall)

Mountfield et al (2014)	**Adherence/ non-adherence, low adherence**	**MMAS-4**	***Low adherence:*** *scores 3-4;*	**21.9%** of participants overall met low adherence criteria
Nahon et al (2011)	**Adherence/ nonadherence**	**Visual Analogues Scale (VAS)**	***Nonadherence:*** *VAS medication adherence rates of <80% (≥80% considered adherent)*	**10.4%** nonadherence to treatment
Nguyen et al (2016)	**Adherence/ nonadherence**	**MMAS-4**	***Low adherence:*** *Higher scores 3-4;*	**21.7%** overall nonadherence across all participants
Ozturk et al (2023)	**Compliance, adherence/ noncompliance, non-compliance**	**MMAS-4**	***Noncompliance:*** *Higher scores 3-4;*	**29.1%** non-compliant patients (as measured by the scale)
Pittet et al (2014)	**Compliance/ noncompliance**	**Study specific developed measure**	***Noncompliance:*** *any of the following categories:*<br>*-"therapy taken according to dosage but not always the frequency,"*<br>*-"therapy taken according to frequency but not always to the dosage,"*<br>*-"therapy taken according neither to dosage nor frequency,"*<br>*-"therapy never taken."*	**19.3%** overall patients were noncompliant
Ramos et al (2021)	**Adherence/ nonadherence, inadequate adherence**	**Modified medication possession ratio (mMPR)**	***Inadequate adherence:*** *<90% mMPR*<br>*(Calculated by comparing the amount of theoretical supply of drug during the follow-up period against the actual amount of drug refill at the outpatient pharmacy service).*	**6.4%** global nonadherence to treatment<br>(Subcut 6.6%; IV NR%)
Ribaldone et al (2017)	**Adherent/ Not-adherent**	**Self-reporting of medication taking in 2 weeks prior to visit**	***Not-adherent****: If participant reported to have missed at least 1 dose of mesalazine in the last 2 weeks.*	**42.6%** not-adherent
Selinger et al (2013)	**Adherence/ non adherence**	**MARS-4**	***Non adherence:*** *Adherence rates <80%*<br>***Scores ranged from: 4-20***<br>***Non-adherers****; scores: 4-16;*<br>***Adherers****; scores: 17-20*	**28.7%** nonadherent to IBD maintenance medication
Severs et al (2017)	**Adherence/ nonadherence**	**Visual Analogue Scale (VAS)**	***Nonadherence:*** *VAS medication adherence rates of <80% (≥80% considered adherent)*	**12.7%** using IBD-specific medication were nonadherent
Shah et al (2020)	**Adherence/ nonadherence**	**MPR**	***Nonadherence:*** *Medication possession ratio of <0.86 (Total sum of days' supply for each medication refill divided by the number of days in the observation period)*	**31%** nonadherent

Stone et al (2021)	**Adherence/ nonadherence**	**MARS-5**<br><br>**Bi-weekly medication tracking**	***Nonadherence:***<br>***MARS-5: Scores ranged from: 5-25***<br>*Standard adherence rates <80%*<br>***Non-adherers***; *scores: 5-20;*<br>***Adherers***; *scores: 21-25*<br>*More rigorous adherence rates <90%*<br>***Non-adherers***; *scores: 5-22.5;*<br>***Adherers***; *scores: 22.5-25*<br>+<br>***Bi-weekly medication tracking:***<br>*Self-reporting by participants of % of each prescribed IBD medication taken over the preceding 2-week period. 2-week % for each IBD medication were averaged over a year to determine overall %.*<br>*(If a participant had multiple IBD medications, all prescribed IBD medications were averaged to give a total medication adherence value).*<br>***Non-adherence defined in rates <90% and rates <80%***	**10.7%** of participants were non-adherent based upon MARS-5 80% cut-off<br><br>**17.9%** of participants were non-adherent based upon a bi-weekly medication tracking cut off of <90%<br><br>**9.8%** of participants were non-adherent based upon a bi-weekly medication tracking cut off of <80%
Suzuki et al (2021)	**Adherence/ non-adherence, nonadherence, low adherence, decreased adherence**	**Study specific developed measure**	***Decreased adherence:*** *categorical variable at the <80% level*	**22.8%** had decreased adherence of <80%
Tae et al (2016)	**Adherence/ nonadherence**	**MMAS-4**	***Low adherence:*** *sum of the scores between 0-2*	**36.2%** assigned to the nonadherent group
Tomar et al (2019)	**Adherence/ nonadherence, non-adherence**	**Study specific developed measure**	***Nonadherence: drug intake <80% of prescribed dose***<br>*(Calculated by adding individual medication adherence rates and dividing by number of medications taken. Patients were then categorised according to adherence rates into 5 categories formed by dividing 0-1005 at 20% intervals).*	**17.7% reported <80% adherence to medication**
van der Have et al (2016)	**Adherence/ non-adherence, low adherence**	**MMAS-8**<br><br>**Pharmacy refills**	***MMAS-8:***<br>*Scores ranged from: 0-8*<br>***Low adherence;*** *scores: <6;*<br>***Medium adherence;*** *scores: 6-7;*<br>***High adherence;*** *scores: 8*<br><br>***Pharmacy refills:*** *Assessed over 12month period. Calculated by dividing number of days supplied within refill interval (based on dosing regimen) by the number of*	**25%** had non-adherence (a MPR <80%),<br>**28%** patients had low adherence (<6 on MMAS-8)

			days in the actual refill interval over 12 months, expressed as %.	
Wang et al (2020)	**Adherence/ nonadherence**	**MARS-4**	***Non adherence:*** *Adherence rates <80%*<br>***Scores ranged from: 4-20***<br>***Non-adherers****; scores: 4-16;*<br>***Adherers****; scores: 17-20*	**41.9%** patients were of AZA nonadherence
Watanabe et al (2021)	**Adherence/ non-adherence**	**Self-administered Visual Analogue Scale (VAS)**	***Non-adherence:*** *taking <80% of their prescribed medication dose, at least once during their pregnancy (≥80% considered adherent)*	**19.2%** average overall adherence self-reported for all medications, with 1st trimester lowest non-adherence levels, (Physician's report under-estimated non-adherence).
Wentworth et al (2018)	**Adherence/ nonadherence**	**Modified medication possession ratio (mMPR)**	***Nonadherence:*** *mMPR <100%*<br>*(calculated by comparing any missed infusion /no-fill medications with expected number of fills according to records over 24 months)*	**34%** overall nonadherence
Yen et al (2012)	**Adherence/ nonadherence**	**MPR or PDC**	***Nonadherence:***<br>***either MPR*** *<0.8*<br>*(Total number of days of index medication supplied/number of days in specified time interval (360 days).*<br>***OR***<br>***Proportion of Days Covered (PDC)*** *<0.8*<br>*(Number of days with any oral 5-ASA drug on hand /number of days in specified time interval (360 days)*	**79%** overall nonadherence rate for any 5-ASA medication
Yoon et al (2017)	**Good compliance/ Poor compliance**	**MMAS**	***Poor compliance:***<br>***Morisky scale score ≥1:*** *Yes to any of 4 questionnaire items*	NR
Yu et al (2019)	**Adherence**	**MMAS**<br><br>**Study Specific Self-report questionnaire** (open+ close-ended questions with 4 sections:<br>1) Sociodemographic data<br>2) IBD-related information +patient preferences	***No cut off reported***	NR

		3) Patients' expectations for online education<br>4) network influence +medication adherence		
Zand et al (2019)	**Adherence/ nonadherence**	**MMAS-8**<br><br>**+ Study Specific Self-report Screening tool for Medication adherence** (2 open-ended questions + 23 closed-ended questions, categorised either as intrinsic, extrinsic or general)	***MMAS-8***<br>***Nonadherent:*** *Score <6*<br>***Adherent:*** *Score ≥6*	**33%** nonadherent
Zelante et al (2014)	**Adherence/ non-adherence, low therapeutic adherence**	**QUOTE-IBD**	***QUOTE-IBD***<br>***No cut off reported (****however classifications of 2 groups of adherent and non-adherent based upon logistic regression analysis and Odds Ratios calculations conducted, to determine adherence to medical treatment).*	**17.35%** reported a non-adherence to therapy

Abbreviations: AZA: azathioprine; IV: intravenous; MARS: Medication Adherence Report Scale; MEMs: Medication Event Monitoring system; MMAS: Modified Morisky Medication Adherence Scale; mMPR: Modified Medication Possession Ratio; MPR: Medication Possession Ratio; NA: Not applicable; PDC: Proportion of Days Covered; pmol: picomoles / red blood cell; NA: Not applicable; NR: Not reported; Subcut: Subcutaneous; TGN: Thioguanine nucleotides, Tx: Treatment.
Key: *Lim et al (2020): Biologic drugs were excluded from adherence analysis.
Note: Terminology used is written as reported in each paper.
