# Supplementary figures and images for: Medication Non-Adherence in Inflammatory Bowel Disease: A Systematic Review Identifying Risk Factors and Opportunities for Intervention
Source: Pharmacy (Basel). 2025 Feb 7;13(1):21. doi: 10.3390/pharmacy13010021 (PMC11859822; doi:10.3390/pharmacy13010021)

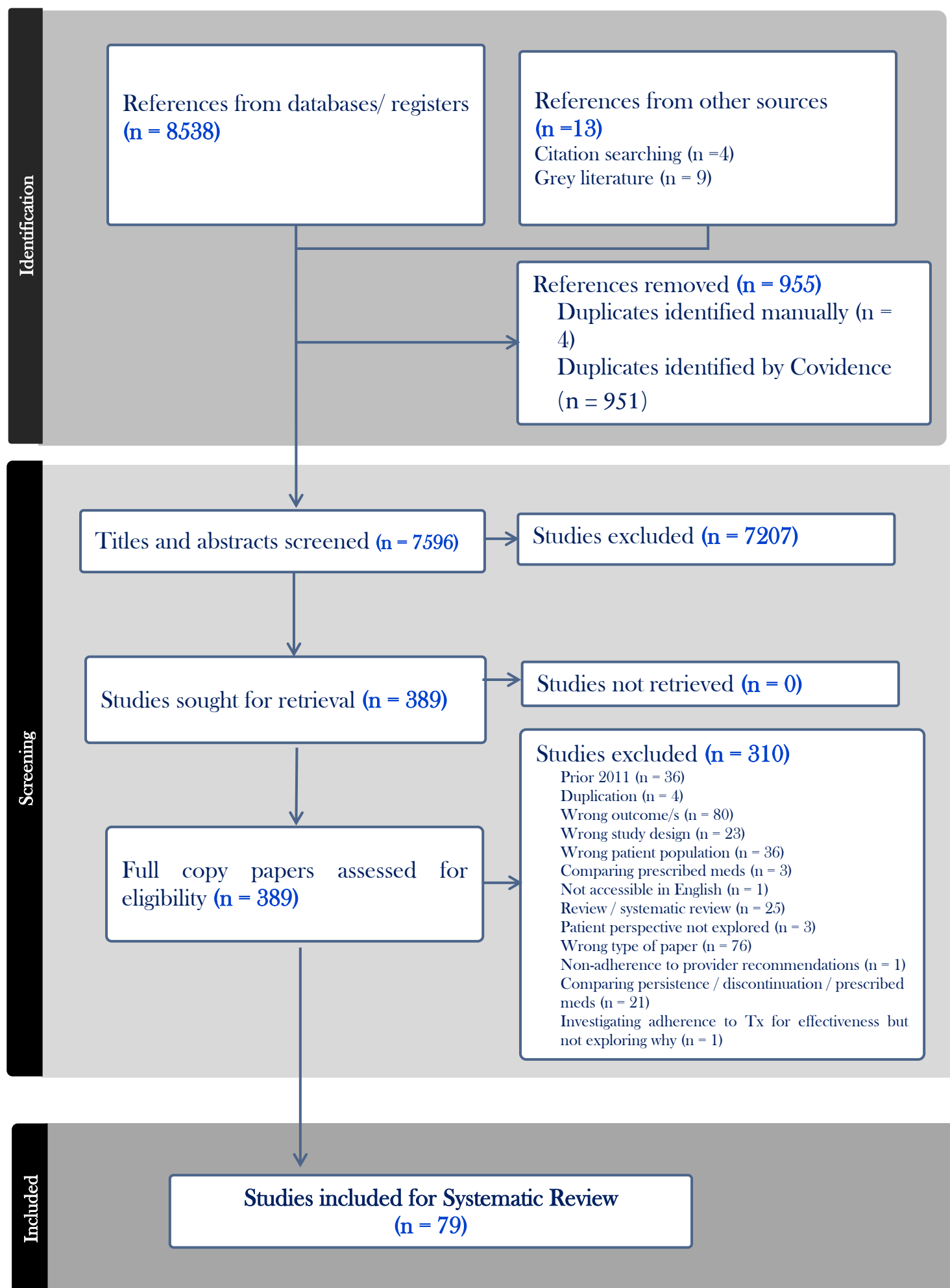

*Supplementary Figure S1: PRISMA Flow Diagram, Selection of studies for Systematic Review*

Supplement: Supplementary file 1 [file pharmacy-13-00021-s001.zip › FINAL Supplementary Figure S1_PRISMA Flow diagram_Systematic Review.pdf]
